# Leadership, teamwork and work outcomes in healthcare: A test of a model

**DOI:** 10.1371/journal.pone.0353609

**Published:** 2026-07-10

**Authors:** Elaine R. Neiva, Ricardo S. S. Durães, Gardênia da Silva Abbad, Andreas Xyrichis

**Affiliations:** 1 Department of Social and Work Psychology, University of Brasília, Brasília, Distrito Federal, Brazil; 2 Florence Nightingale Faculty of Nursing, Midwifery and Palliative Care, King’s College London, London, United Kingdom; STIKES Wira Medika PPNI Bali: Sekolah Tinggi Ilmu Kesehatan Wira Medika PPNI Bali, INDONESIA

## Abstract

Effective teamwork in healthcare improves patient outcomes, staff well-being, and service delivery. To enhance these benefits, NHS teams should implement targeted leadership training, routinely assess and clarify roles to reduce ambiguity, and encourage staff participation in decision-making to boost autonomy. Researchers analysed 2022 and 2023 NHS Staff Survey data using Structural Equation Modelling. Main findings: (1) Effective leadership boosts teamwork and, in turn, engagement and well-being; (2) Teamwork can raise role ambiguity and lessen autonomy, leading to stress; (3) Leadership is key to managing these issues. Thus, boosting autonomy and reducing ambiguity support better team performance and staff well-being. The study highlights the need for leadership programmes to strengthen team dynamics and reduce uncertainty.

## Introduction

Teams rather than individuals deliver modern health care and require the cooperation of health care professionals from multiple disciplines [1]. Interprofessional healthcare teams rely on effective teamwork and communication to ensure safe and effective patient care [2–4]. The use of teams has grown significantly in healthcare organisations, becoming a fundamental part of care delivery. Approximately 60% of primary care practices in the United States use team-based models [5]. The percentage can approach 100% in many other countries. In the UK, for example, interprofessional teams integrating services across general practice, community health, and social care is key within the National Health Service (NHS) long-term plan [6], as is workforce reform to foster more effective teamwork [7].

The NHS Staff Survey is a comprehensive annual assessment of NHS staff experiences, conducted since 2003. Each year, NHS staff are invited to participate, providing a broad perspective on workplace satisfaction and conditions. By delivering both national overviews and local insights, the survey enables organisations to identify challenges and develop targeted solutions, thereby supporting collaborative efforts to enhance the NHS. In 2021, the survey content aligned with the NHS People Promise, which seeks to foster an inclusive and compassionate culture in which staff are recognised, rewarded, empowered, continuously learning, able to work flexibly, and integrated into effective teams. Additionally, survey results are a key resource for monitoring the implementation of the NHS Long Term Workforce Plan. This plan aims to strengthen the health system through digital and technological innovation, improved workforce planning, expanded and reformed education and training, optimisation of multidisciplinary teams, and the development of workforce skills to leverage technological advancements. The plan also promotes the adoption of innovative working practices.

Daniels et al. [8] report that the pandemic resulted in work overload, increased retirements, and illness among NHS staff. The overall sickness absence rate for NHS staff in England rose to 5.7% in October 2021, compared to 4% in June 2020 [9]. High staff vacancy and turnover rates have also been observed. Multiple studies indicate that poor health and well-being among NHS staff are linked to reduced quality of care, diminished financial performance, and lower patient satisfaction. This narrative review estimates that poor well-being among NHS staff costs the NHS £12.1 billion annually, and suggests that approximately £1 billion could be saved through effective interventions.

According to Wallbank [10], the results of the NHS Survey, published in 2022, showed a worrying situation. Staff expressed dissatisfaction with salaries, and workforce morale was declining. About a third of respondents considered leaving the organization, an increase of 5.7 percent since 2020 (The NHS Staff Survey [11] 2022: What Do The Results Tell Us? 2023). The results showed ongoing annual deterioration, with 32.3 percent of staff considering leaving. Nursing staff, healthcare assistants, and ambulance staff reported the highest dissatisfaction and intended to leave as soon as they found another job. The intention to leave is now more likely to be acted upon than in previous years. Career abandonment in favor of roles outside the NHS has become a considered alternative.

The 2023 NHS Staff Survey [12], completed by almost 700,000 professionals across clinical, non-clinical, and care settings, showed small improvements in some scores compared to 2022. There was a small reduction in reports of burnout, sick leave, or staff coming into work sick, and a modest reduction in staff considering leaving the service. However, these improvements may not persist and could return to pre-pandemic levels. Performance problems, such as patient care waiting times and clinical workforce attrition rates, still persist, according to Oliver [13].

Only 44% of participants reported believing that the organisation values their work. Although two-thirds felt valued by their own peers or had a strong connection to their team. More than 70% felt heard and supported by their immediate manager. Approximately 30% of respondents reported feeling exhausted by their work, 34% reported it was emotionally draining, but only 57% reported that their organization had taken positive steps toward health and well-being, according to Oliver [13].

Less than half of those interviewed felt able to satisfy the conflicting demands of their work. Only a third said their workplace had enough staff to do their job properly, and only a quarter said they never faced unrealistic time pressures. Despite ongoing problems, high professional commitment remains evident. Approximately 90% believe their role makes a difference to patients, and 70% say patient care is their organization’s top priority, according to Oliver [13].

A longitudinal study by Moscelli et al. [14] found that nurse and doctor engagement is positively associated with managers’ use of effective communication, staff involvement in decision-making, and responsiveness to feedback. Notably, older nurses’ engagement increases when managers prioritize staff health and well-being. Their findings show that nurse engagement is associated with increased nurse retention, and that higher nurse retention is linked to higher physician retention through retaining more experienced nurses. Leadership and peer social support contribute to improving relevant outcomes.

To address these challenges and improve NHS well-being, health, and patient care indicators, it is recommended that we investigate which job and personal resources—such as professional development opportunities, access to supportive supervision, and flexible work schedules—most effectively improve staff work processes and outcomes. Such empirical investigation could contribute to the success of the ongoing national action plan. Leadership behaviours, teamwork, and other job and personal-related factors should also be prioritized, as they are positively associated with well-being, efficiency, retention, and quality indicators of patient care.

While the importance of teamwork in healthcare is well-established [15–19], existing literature often lacks a comprehensive theoretical integration of how leadership improves team dynamics and how specific job characteristics, such as autonomy, a job resource, and managing conflicting job demands skills, a personal resource, mediate these relationships to impact employee well-being and engagement. This study addresses this gap by applying the Job Demands-Resources (JD-R) model to investigate the complex relationships between leadership, teamwork, and work outcomes within healthcare teams in England’s NHS, providing a more nuanced understanding of these pathways. Furthermore, these relationships are tested using data from 2022 and 2023.

### Background

The term “teamwork” encompasses a wide range of behavioural processes that individuals utilise to carry out interdependent work, along with the affective and cognitive states that emerge during such tasks [20]. Behavioural processes include communication, coordination, drawing on others’ expertise, and mutual aid. Emergent states include, for example, mutual respect and psychological safety. Behavioural processes and emergent states are distinct from enduring traits, group structures, individual characteristics, and task work (e.g., interactions with tools and systems) [21]. Following a concept analysis, Xyrichis and Ream [15] defined teamwork in health care as “a dynamic process involving two or more health care professionals with complementary backgrounds and skills, sharing common health care goals, and exerting focused physical and mental effort in assessing, planning, or evaluating patient care.”

Teamwork is also an important predictor of another indicator of hospitals’ organizational performance: the well-being of healthcare providers [22,23]. Reduced occupational well-being or high psychological strain may develop as an immediate or long-term response to stressors [24] and is highly prevalent in healthcare workers [25,26]. Teamwork may constitute such a stressor. For instance, dysfunctional inter-professional teamwork predicts increased acute and chronic clinician strain [27,28]. However, effective teamwork may protect team members from the effects of work stress, since positive perceptions of teamwork are associated with enhanced occupational well-being indicators such as better mental health in nurses and physicians [29,30].

Research suggests that the benefits of effective teamwork can be substantial. Studies show better team functioning is associated with improved performance [16], patient outcomes [17–19], and cost savings [31]. Researchers have theorised that these benefits accrue because more effective teams make better decisions, handle complex tasks more effectively, produce more integrated care plans based on combined expertise, and coordinate their actions more effectively [31–34].

Working in autonomous teams has been linked to various organisational effectiveness outcomes, including patient mortality [35], performance outcomes, and team member attitudes such as engagement (e.g., [36,37]). Similar conclusions were reached in Applebaum and Batt’s [38] review of 12 large-scale surveys and 185 case studies of management practices. The authors concluded that teamwork (not necessarily autonomous) improves organisational performance regarding efficiency and quality measures. In a qualitative review of 31 studies, Delarue et al. [39] concluded that teamwork positively impacts four dimensions of performance outcomes (operational, financial, attitudinal, and behavioural outcomes).

To understand the relationship between the work situation, its factors, and its consequences on engagement, well-being, and productivity, this study is based on The Job Demands and Resources Model – JD-R. The Job Demands and Resources Model is an organizational psychology framework explaining how job characteristics influence employee well-being, engagement, and performance. It posits that high job demands (e.g., workload) cause burnout, while abundant job resources (e.g., autonomy, support) foster engagement, making it a tool to balance workload with resources to improve health and productivity. Within the JD-R model, work characteristics are broadly categorized into job demands and job resources, which influence employee well-being and performance through distinct processes. This framework provides a robust theoretical lens for understanding the complex interplay of factors in healthcare settings.

Job Demands are defined as physical, psychological, social, or organizational aspects of the job that require sustained effort and are associated with physiological and psychological costs [40–42]. Job Resources are those aspects of the job that are functional in achieving work goals, reducing job demands and their associated costs, or stimulating personal growth and development [43,44]. In this study, leadership, teamwork, and autonomy are considered crucial job resources. Effective leadership behaviors, such as providing support and clear feedback, serve as vital resources that can shape the work environment and provide additional resources [45,46]. Teamwork, as a social resource, fosters community, social interaction, and cohesion, enhancing a sense of belonging and facilitating goal achievement [40,43].

Autonomy in the workplace is a well-established job resource. It provides individuals with control and influence over their work, reducing stress and increasing productivity. The JD-R model posits that job resources are key predictors of work engagement and might buffer the negative effects of job demands on well-being [43,44].

Among personal resources, self-efficacy and optimism are aspects that favor a balance between work demands and resources. In the NHS Staff Survey, coping with ambiguity refers to an individual resource, defined as the skills to manage conflicting work demands, and is measured by two items assessing whether the professional can manage such demands in the workplace. Within the Job Demands–Resources (JD-R) model, coping with ambiguity at work can be conceptualized as a regulatory process through which employees manage task and role ambiguity, a core job demand associated with uncertainty and contradictory expectations. Task ambiguity undermines work engagement and well-being, whereas behaviors that reduce ambiguity, such as managing up, function as demand-reducing mechanisms that indirectly enhance engagement by clarifying role expectations [47].

Work engagement is a positive outcome, a fulfilling, work-related state of mind characterized by vigor, dedication, and absorption. It is a key outcome of the motivational process driven by job resources within the JD-R model.

Well-being is another variable, often defined in the JD-R model as the absence of burnout or strain, reflecting a health-impairment process driven by high job demands. The items used in this study to measure well-being reflect this negative dimension, capturing discomfort and psychological distress.

### Study hypotheses

Based on the JD-R model, we propose the following hypotheses that outline the expected causal relationships among leadership, teamwork, autonomy, ambiguity, work engagement, and well-being in healthcare settings. The JD-R model posits that job resources (e.g., leadership, teamwork, autonomy) foster a motivational process related to engagement and positive outcomes, whereas conflicting and excessive job demands initiate a health-impairment process associated with strain and negative well-being [43,44].

**H1.** *There is a positive relationship between teamwork and work outcomes (engagement and well-being).*

*Rationale.* Within the JD-R model, teamwork functions as a crucial social job resource. Resources are known to fuel motivational processes, leading to higher work engagement and improved well-being by facilitating objective achievement and fostering a supportive work environment [43,44,46].

**H2.** *There is a positive relationship between teamwork and well-being at work.*

*Rationale.* As a job resource, effective teamwork contributes directly to employee well-being by providing social support and reducing the impact of job demands. However, the JD-R model also acknowledges that if teamwork introduces new demands (e.g., through role ambiguity) or fails to provide sufficient resources (e.g., autonomy), its net effect on well-being may be complex [41,48,49].

**H3.** *The relationship between teamwork and well-being at work is mediated by work engagement.*

*Rationale.* According to the JD-R model, job resources (like teamwork) are key predictors of work engagement, which is a motivational state. This engagement, in turn, acts as a protective factor that might mitigate the negative effects of job demands and enhance overall well-being [50–52].

**H4.** *Leadership is directly associated with effective teamwork.*

*Rationale.* Leadership is a critical job resource within the JD-R framework. Effective leaders provide the necessary support, structure, and vision that enable and foster high-quality teamwork, aligning team goals with organizational objectives [45,46].

**H5.** *Leadership is associated with teamwork in a relationship mediated by autonomy.*

*Rationale.* As a job resource, leadership can directly associate the availability of other resources, such as autonomy. When leaders promote autonomy, they empower team members to take ownership and make decisions, thereby making this job resource a facilitator of more effective, self-directed teamwork, consistent with the JD-R model’s motivational pathway [43–45].

**H6.** *Leadership is associated with teamwork in a relationship mediated by perceived coping with ambiguity.*

*Rationale.* Leadership plays a crucial role in managing job demands within the JD-R model. By reducing role ambiguity (a significant job demand), leaders create a clearer and less stressful work environment. This reduction in demands, in turn, facilitates more effective teamwork by minimizing confusion and enhancing coordination [40,42,53].

This study tests these associations within the Job Demands-Resources model, considering how leadership is associated with teamwork through the mediation of autonomy (a resource) and coping with ambiguity (another resource), and how these, in turn, affect perceptions of well-being and engagement. These questions are important because prior research suggests that the quality of supervision affects subordinates’ access to information, resources, and support, thereby influencing the quality of teamwork and, consequently, how workers address task ambiguity, which can lead to “near misses”/errors. Furthermore, although research links leadership to perceptions of well-being, it is unclear whether leadership affects teamwork and whether these perceptions, in turn, affect nurses’ well-being. Therefore, this study aims to examine the relationships among leadership, teamwork, and work outcomes in professional teams, using data from the NHS in England.

The JD-R framework further posits that personal resources play a central role in coping with demanding work conditions. Personal resources such as resilience and self-regulatory capacity support employees’ ability to cope with ambiguity by buffering the strain associated with unclear roles and sustained uncertainty [54,55]. When these resources are depleted, employees become less capable of managing ambiguity, increasing susceptibility to stress and disengagement [56,57].

Coping strategies represent mechanisms through which individuals mobilize resources in response to ambiguity. Problem-focused control coping aligns with the JD-R assumption that proactive resource investment mitigates job demands, whereas reliance on emotion-focused support coping may indicate prolonged exposure to unresolved demands that exceed available resources [58]. From this perspective, coping with ambiguity reflects the dynamic interplay between job demands, personal resources, and regulatory strategies, consistent with the core assumptions of the JD-R model.

## Materials and methods

### Participants

The sample was obtained from the NHS National Staff Survey, an annual survey conducted across NHS organizations in England to gather insights into the experiences and opinions of NHS employees. It serves as a key instrument for understanding workforce well-being, engagement, and satisfaction, offering critical data to inform policy and organisational improvements. The survey measures various aspects of staff experience, including workplace culture, leadership, team dynamics, job satisfaction, and the availability of resources to deliver quality care. The data were analysed at the group level, with percentage agreement calculated for each group. The study included 1,210 respondents, with 600 surveyed in 2022 and 610 in 2023. These respondents were aggregated into 564 professional groups, representing diverse roles across various NHS trusts. The aggregability of contextual variables was verified using Intraclass Correlation Coefficients (ICC) and the Median Absolute Deviation (MAD). The ICCs presented high and statistically significant values (*p* < 0.001), between 0.712 and 0.898 for single measures and between 0.832 and 0.946 for means, indicating high intragroup consistency. The MAD was calculated for all participating teams, yielding a mean of 0.44 (*SD* = 0.32), with a cutoff of 0.83 for teams that remained below the critical value. This indicates that cognitive sharing was observed in the vast majority of teams.

To complement these indices, a one-way analysis of variance (ANOVA) was conducted for each study variable to verify the presence of meaningful between-group variance, a necessary condition for justifying aggregation to the group level [59]. The ANOVA results confirmed statistically significant between-group variability across all constructs (*p* < 0.001), supporting the existence of systematic group-level effects and confirming that the variance structure of the data is consistent with a multilevel representation of the constructs. Regarding within-group agreement, the Median Absolute Deviation (MAD) was adopted as the primary index rather than the more traditional r_wg(j)_. This choice is methodologically grounded in well-established recommendations in the interrater agreement literature. The MAD is conceptually analogous to the Average Deviation (AD) index proposed by Burke, Finkelstein, and Dusig [60] and has been recommended as a robust alternative to r_wg(j)_ for three main reasons [61–63]. First, unlike r_wg(j)_, which depends on an assumed null distribution (typically rectangular) and can yield uninterpretable values outside the 0–1 range or negative values that must be truncated, the MAD is a distribution-free index expressed directly on the original metric of the response scale, facilitating a more transparent interpretation of the degree of agreement among respondents. Second, the MAD is less sensitive to variations in group size and rating scale characteristics, which is particularly relevant in our study given the heterogeneity of professional groups and the variable number of respondents within each NHS trust. Third, the MAD provides a direct measure of the typical deviation of individual responses from the group central tendency, which is conceptually aligned with the construct of within-group agreement and more intuitive for applied audiences. For a five-point Likert scale, the recommended cutoff is 0.83 [60–63], a criterion met by the vast majority of teams in our sample. Taken together, the convergence of statistically significant between-group ANOVA results, high and statistically significant ICC_(1)_ and ICC_(2)_ values, and MAD values below the recommended cutoff provides robust empirical and methodological justification for aggregating the data to the group level [59–63]. Benchmarking groups for 2022 and 2023 included: Acute and Combined Acute & Community Trusts; Acute Specialist Trusts; Mental Health & Learning Disability Trusts; Combined Mental Health & Learning Disability and Community Trusts; Community Trusts; and Ambulance Trusts. While specific individual-level demographic data (e.g., age, gender, years of experience) were not accessible due to the anonymized nature of the secondary data, these groups encompass a wide range of healthcare professionals, including nurses, doctors, allied health professionals, and administrative staff, reflecting the multidisciplinary nature of NHS teams. The 2022 data were analysed for their timeliness and relevance to test the model.

### Ethics statement

This study utilised secondary data obtained from the NHS National Staff Survey, originally collected by NHS England for workforce monitoring and organisational improvement. The data were fully anonymised before access and analysis, ensuring that no identifiable information was available to the researchers. The primary data collection was reviewed by a Research Ethics Committee (REC) under the UK Research Ethics Service, which operates within the Health Research Authority (HRA) and the Devolved Administrations to safeguard participants’ rights, safety, dignity, and well-being. Additionally, informed consent was obtained from participants at the time of data collection for the use of their responses in research. In accordance with HRA guidelines and the Governance Arrangements for Research Ethics Committees (GAfREC) in the United Kingdom, further ethical approval was not required for this study, as it involved the analysis of pre-existing, fully anonymised data [64].

### Measures

We used items from the NHS Staff Survey to evaluate key variables, as presented below. The items used in this research were selected by three judges, who assessed their content validity and alignment with the theoretical frameworks. We also analysed the items’ content and the results of the statistical factor analysis to develop the model for testing.

**Teamwork.** The NHS Teamwork Scale comprises nine items, rated on a five-point Likert scale ranging from 1 (Disagree) to 5 (Agree). An example of an item is: I feel valued by my team. Items 7a to 7i have been used for this model test purposes.

**Work Engagement.** This model test used three items (2a, 2b, and 2c) to evaluate work engagement. Examples of items are: Q2a - *I look forward to going to work*; Q2b - *I am enthusiastic about my job*; Q2c - *Time passes quickly when I am working*.

**Leadership.** The leadership items correspond to questions 9a-9h of the NHS questionnaire. Examples: Q9a - *My immediate manager (who may be referred to as your ‘line manager’) encourages me at work*; Q9b - *My immediate manager (who may be referred to as your ‘line manager’) gives me clear feedback on my work*.

**Autonomy.** The items on autonomy comprise questions 3c, 3d, 3e, and 3f in the NHS questionnaire. Examples: Q3c - There are frequent opportunities for me to show initiative in my role; Q3d - *I am able to make suggestions to improve the work of my team/department*; Q3e - *I am involved in deciding on changes introduced that affect my work area/team/department*; Q3f - *I am able to make improvements happen in my area of work*.

**Well-being.** The well-being items assess how often professionals experience discomfort and illness at work, using a five-point frequency scale ranging from 1 (never) to 5 (always). These items, such as “*How often, if at all, do you find your work emotionally exhausting?*” (Q12a), “*How often, if at all, does your work frustrate you?*” (Q12c), and “*How often, if at all, do you feel that every working hour is tiring for you?*” (Q12f), effectively capture aspects of psychological distress and ill-being. This focus on negative indicators of well-being aligns with the JD-R model’s health impairment process, which examines the costs associated with job demands and their impact on strain and burnout. While these items provide valid insights into the absence of well-being, future research could complement these findings by incorporating measures of positive well-being to provide a more integral view.

**Coping with ambiguity.** In the NHS Staff Survey, coping with ambiguity refers to an individual resource, defined as the skills to manage conflicting work demands, and is measured by two items assessing whether the professional can manage such demands in the workplace. The two ambiguity items assess whether the professional can deal with conflicting demands in the workplace. An example of an item is: “*I am able to meet all the conflicting demands on my time at work. I am able to deal with contradictory demands at work*.” While these two items indicate the extent to which staff workers deal with task ambiguity. Although there are only two items, the confirmatory factor analysis of the measurement model yielded factor loadings above 0.50 and reliability indices above 0.75 (Cronbach’s Alpha and McDonald’s Omega). Specifically, both items showed high standardised loadings on the latent factor (consistent with an Average Variance Extracted of 0.872, implying average loadings above 0.90), a Cronbach’s Alpha of 0.893, and McDonald’s Omega values ranging from 0.912 to 0.914 across estimators, all substantially exceeding the conventional thresholds of 0.70 for reliability and 0.50 for convergent validity [65]. These indicators are reported for ambiguity coping in Table 6, together with the other latent constructs. Beyond the psychometric adequacy of the scale, the construct validity of the two-item measure is further supported by three conceptual considerations. First, the scale was designed to capture a narrowly defined construct, coping with task ambiguity, understood as the perceived capacity to manage conflicting or contradictory work demands, rather than the broader and multidimensional role ambiguity construct [66]. When a construct is conceptually narrow and the items are content-valid for that specific domain, short scales can achieve acceptable reliability and validity, provided that inter-item correlations are high and the items adequately represent the construct [67,68]. Second, methodological research demonstrates that two-item measures, although not optimal, can yield psychometrically sound indicators when the items are well-constructed, conceptually homogeneous, and empirically correlated, and that their use is particularly justified in large-scale workforce surveys where questionnaire length must be balanced against participation rates [69,70]. Third, the items of the NHS Staff Survey have been developed through extensive consultation, cognitive testing, and iterative refinement over more than two decades of annual administration, supporting their content validity for the UK healthcare workforce. While we acknowledge that a multi-item scale capturing distinct facets of task and role ambiguity would represent an improvement, a limitation explicitly discussed later in this paper, the convergent psychometric evidence (loadings, reliability, AVE) and the theoretical delineation of the construct support its use for testing the hypothesised model.

### Procedures

The NHS National Staff Survey provides aggregated data to preserve respondents’ anonymity and comply with data governance requirements. As one of the most extensive workforce surveys globally, it is conducted annually to enhance staff experiences across the NHS. To address the absence of individual-level data and to focus on organizational-level dynamics, we used group-level data. This approach involved analysing data from 564 professional groups (e.g., Acute, Mental Health, Community Professionals), each comprising more than 11 cases, ensuring representative data within each group. This group-level analysis enables examination of broader organizational trends and team-level phenomena, aligning with the study’s focus on leadership and teamwork within healthcare teams. While this approach precludes individual-level inferences and carries the risk of ecological fallacy, it was a necessary methodological choice given the nature of the secondary data and enables a robust analysis of team-level constructs within a large national dataset.

### Statistical analysis

We decided to analyse the data at the group level using a percentage of agreement/frequency, but we also used the disagreement rate to verify the result’s consistency. We obtained evidence of validity from the Exploratory Factor Analysis (EFA) conducted on 2021 data, which empirically explored and established the underlying factorial structure of the selected scales (Leadership, Teamwork, Engagement, Well-being, Autonomy, Coping with Ambiguity). The results of this EFA then guided the specification of the measurement model for the Confirmatory Factor Analysis (CFA) performed on the 2022 data. The CFA was conducted to validate the hypothesized factorial structure and assess the reliability and validity of the measures before proceeding with the full Structural Equation Modeling (SEM) analysis. We calculated some other reliability measures (Cronbach’s Alpha, McDonald’s Omega, Average Variance Extracted, and Composite Reliability) using both 2021 and 2022 data. Structural Equation Modeling was performed using Robust Diagonally Weighted Least Squares (RDWLS). RDWLS is a robust estimation method particularly suitable for structural equation modeling with ordinal or categorical data (such as the Likert scales from the NHS Staff Survey) and for large sample sizes, as it does not assume multivariate normality and provides robust standard errors and chi-square statistics, which is appropriate for the nature of the NHS Staff Survey data. Statistical analysis was performed using the Factor Analysis software (version 12.04.05) and JAMOVI software (version 2.5.6).

## Results

The model was tested using data from 2022 and 2023, yielding very similar adjustment indices across the two periods. The multigroup analysis also presented adjustment indices consistent with those obtained previously. Table 1 presents the model fit indices for the structural equation models tested with the 2022 and 2023 datasets, as well as the multigroup analysis. Across all models, the Comparative Fit Index (CFI), Tucker–Lewis Index (TLI), and Normed Fit Index (NFI) met or exceeded the commonly accepted threshold of 0.90, indicating an acceptable to good fit. The Standardized Root Mean Square Residual (SRMR) values were below 0.08 in all cases, further supporting adequate model fit.

**Table 1:** Model fit indices.

Year	χ²	*df*	CFI	TLI	NFI	SRMR	RMSEA
2022	1581	123	0.92	0.9	0.91	0.044	0.145
2023	3005	161	0.91	0.9	0.91	0.053	0.152
Multigroup Analysis	2895	157	0.92	0.9	0.91	0.046	0.132

CFI: Comparative Fit Index (acceptable values typically > 0.90); TLI: Tucker-Lewis Index (acceptable values typically > 0.90); NFI: Normed Fit Index (acceptable values typically > 0.90); SRMR: Standardized Root Mean Square Residual (acceptable values typically < 0.08); RMSEA: Root Mean Square Error of Approximation (values < 0.08 generally indicate good fit, although higher values may be acceptable for complex models or very large samples, as discussed in the Results section).

The Root Mean Square Error of Approximation (RMSEA) values were higher than the conventional cutoff of 0.08 for both years. However, given the complexity of the model and the large sample sizes, these values were considered acceptable and consistent with recommendations in the structural equation modeling literature. Overall, the fit indices suggest that the hypothesized model provides a satisfactory representation of the data for both years and in the multigroup analysis.

While the residual indices (RMSEA) were high (0.145 for 2022, 0.152 for 2023, and 0.132 for the multigroup analysis), it is important to note that for complex models with a large number of observed variables and/or very large sample sizes (as is the case with aggregated data from 1,210 respondents across 564 groups), RMSEA values can be inflated and may not be the sole indicator of model fit. Other fit indices, such as CFI, TLI, and NFI (all above 0.90), and SRMR (below 0.08), collectively indicate a reasonable model fit despite the elevated RMSEA.

When considering the relationships between the model variables, the main direct predictive relationships were confirmed by the sample data. Specifically, group work was a significant predictor of key work outcomes, including engagement and well-being. The observed negative relationships between group work and well-being, as indicated by the measures, are attributable to the nature of the well-being items, which describe illness and physical and psychological discomfort. This aligns with the JD-R model’s focus on the health impairment process, in which high demands or insufficient resources can lead to negative well-being outcomes, even in the presence of positive resources such as teamwork.

### Direct effects (2022)

**Fig 1:**
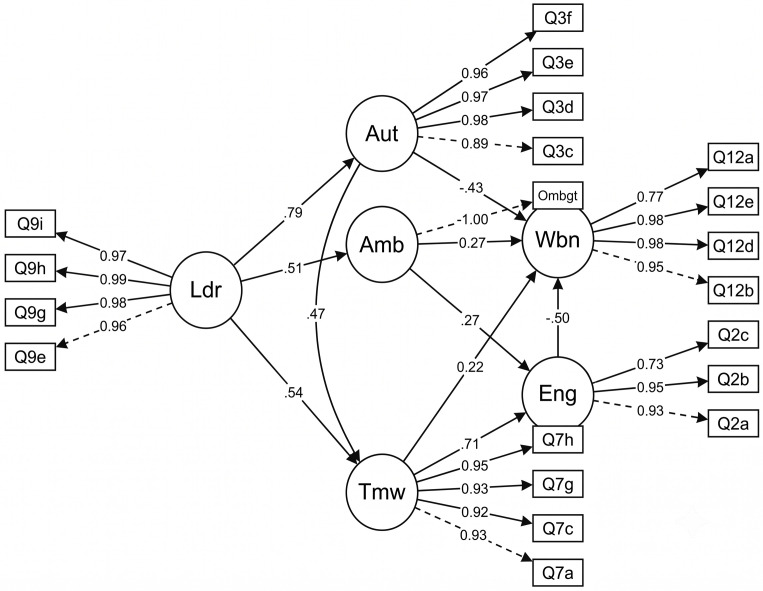
Graphs of the model tested with data from 2022. Ldr: leadership; Aut: autonomy; Amb: ambiguity coping; Tmw: teamwork; Wbn: well-being; Eng: engagement. Note: This model illustrates the hypothesized relationships among leadership, autonomy, ambiguity, teamwork, engagement, and well-being within NHS healthcare teams, according to the Job Demands-Resources (JD-R) model. All reported paths represent significant relationships (*p* < 0.05). Model fit indices are provided in Table 1. Straight lines indicate significant relationships at the *p* <0.05 level. Dashed lines indicate non-significant relationships. The indirect effect is calculated by multiplying the path coefficients of the individual direct paths.

As shown in Table 2 and illustrated in Fig 1, all hypothesized direct paths in the 2022 model were statistically significant (*p* < .05). Engagement was positively predicted by teamwork (β = 0.383, *p* < .001) and coping with ambiguity (β = 0.605, *p* < .001), with ambiguity showing a particularly strong effect.

**Table 2:** Direct predictive relationships between variables with 2022 data.

DV	Predictor	Estimate	SE	*β*	Z	*p*	95% CI	
							Lower	Upper
Engagement	Teamwork	0.332	0.039	0.383	8.50	<.001	0.041	0.383
Engagement	Ambiguity	0.598	0.041	0.605	14.54	<.001	0.678	0.605
Well-being	Teamwork	0.204	0.097	0.190	2.10	<.05	0.393	0.190
Well-being	Autonomy	−0.373	0.074	−0.368	−5.05	<.001	−0.228	−0.368
Well-being	Engagement	−0.203	0.105	−0.164	−2.94	<.05	−0.408	−0.003
Well-being	Ambiguity	−0.776	0.088	−0.635	−8.76	<.001	−0.949	−0.602
Teamwork	Autonomy	0.452	0.051	0.477	8.92	<.001	0.552	0.477
Teamwork	Leadership	0.443	0.051	0.502	8.63	<.001	0.342	0.544
Ambiguity	Leadership	0.547	0.035	0.707	15.42	<.001	0.616	0.707
Autonomy	Leadership	0.827	0.032	0.889	26.18	<.001	0.889	0.889

DV: dependent variable. Estimate: Unstandardized Regression Coefficient; SE: Standard Error; β: Standardized Beta Coefficient (indicating effect size); Z: Z-score for significance test.

Well-being was positively associated with teamwork (β = 0.190, *p* < .05) but negatively associated with autonomy (β = −0.368, *p* < .001), engagement (β = −0.164, *p* < .05), and coping with ambiguity (β = −0.635, *p* < .001). Among these predictors, coping with ambiguity demonstrated the strongest negative relationship with well-being.

Leadership emerged as a key antecedent variable, exerting strong positive effects on autonomy (β = 0.889, *p* < .001), ambiguity coping (β = 0.707, *p* < .001), and teamwork (β = 0.502, *p* < .001). Autonomy also positively predicted teamwork (β = 0.477, *p* < .001), reinforcing its role as a job resource within the model.

Leadership was also a predictor of group work, insofar as it made such work possible. The measures of effect intensity were very similar when comparing data from 2022 and 2023. These results are in Tables 2 and 3.

**Table 3:** Direct predictive relationships between variables with 2023 data.

DV	Predictor	Estimate	SE	*β*	Z	*p*	95% CI	
							Lower	Upper
Engagement	Teamwork	0.748	0.0326	0.709	22.97	<.001	0.6844	0.812
Engagement	Ambiguity	0.239	0.0243	0.267	9.83	<.001	0.191	0.286
Teamwork	Leadership	0.465	0.0237	0.536	19.6	<.001	0.4186	0.512
Teamwork	Autonomy	0.432	0.026	0.470	16.6	<.001	0.3806	0.483
Ambiguity	Leadership	0.520	0.0374	0.508	13.89	<.001	0.4462	0.593
Autonomy	Leadership	0.745	0.0306	0.788	24.31	<.001	0.6846	0.805
Well-being	Teamwork	0.228	0.0759	0.217	3.00	<.01	0.0789	0.376
Well-being	Autonomy	−0.417	0.0552	−0.434	−7.55	<.001	−0.5249	−0.309
Well-being	Ambiguity	−0.238	0.0266	−0.268	−8.95	<.001	−0.2901	−0.186
Well-being	Engagement	−0.498	0.0539	−0.502	−9.25	<.001	−0.604	−0.393

DV: dependent variable. Estimate: Unstandardized Regression Coefficient; SE: Standard Error; β: Standardized Beta Coefficient (indicating effect size); Z: Z-score for significance test.

### Direct effects (2023)

**Fig 2:**
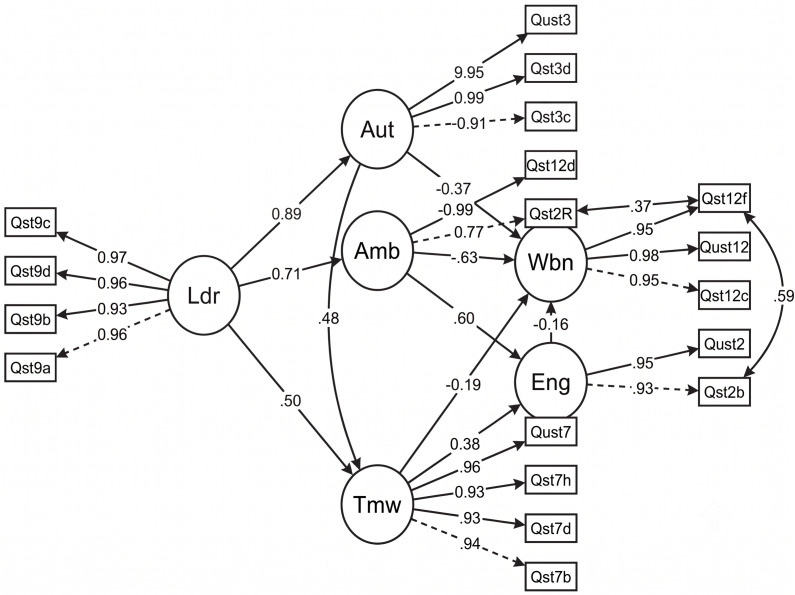
Graphs of the model tested with data from 2023. Ldr: leadership; Aut: autonomy; Amb: coping with ambiguity; Tmw: teamwork; Wbn: well-being; Eng: engagement. Note: This model illustrates the hypothesized relationships between leadership, autonomy, coping with ambiguity, teamwork, engagement, and well-being in NHS healthcare teams, according to the Job Demands-Resources Model (JD-R). All reported paths represent significant relationships (*p* < 0.05). Model fit indices are provided in Table 1. Straight lines indicate significant relationships at the *p*<0.05 level. Dashed lines indicate non-significant relationships. The indirect effect is calculated by multiplying the path coefficients of the individual direct paths.

Table 3 and Fig 2 present the results for the 2023 dataset. The pattern of relationships was largely consistent with the 2022 findings, with all specified paths reaching statistical significance (*p* < .01).

Engagement was again positively predicted by teamwork (β = 0.709, *p* < .001) and coping with ambiguity (β = 0.267, *p* < .001), although the effect of teamwork on engagement was notably stronger in 2023. Well-being showed positive associations with teamwork (β = 0.217, *p* < .01) and negative associations with autonomy (β = −0.434, *p* < .001), coping with ambiguity (β = −0.268, *p* < .001), and engagement (β = −0.502, *p* < .001).

Leadership continued to display strong positive effects on autonomy (β = 0.788, *p* < .001), coping with ambiguity (β = 0.508, *p* < .001), and teamwork (β = 0.536, *p* < .001). Autonomy remained a significant predictor of teamwork (β = 0.470, *p* < .001), replicating the resource-building pathway observed in 2022.

### Indirect effects (2022)

Table 4 reports the significant indirect effects identified in the 2022 model. Coping with ambiguity exerted a substantial negative indirect effect on well-being through engagement (β = −0.256, *p* < .001), as well as through a longer pathway involving teamwork and engagement (β = −0.042, *p* < .001).

**Table 4:** Indirect effects between variables with 2022 data (significant effects).

Parameter	Estimate	SE	*β*	Z	*p*
Ambiguity ⇒ Engagement ⇒ Well-being	−0.245	0.028	−0.256	−8.629	<.001
Ambiguity ⇒ Teamwork ⇒ Engagement ⇒ Well-being	−0.04	0.005	−0.042	−7.526	<.001
Teamwork ⇒ Engagement ⇒ Well-being	−0.498	0.044	−0.448	−11.352	<.001
Autonomy ⇒ Teamwork ⇒ Engagement ⇒ Well-being	−0.34	0.032	−0.326	−10.49	<.001
Leadership ⇒ Teamwork ⇒ Engagement ⇒ Well-being	−0.101	0.014	−0.105	−7.129	<.001
Leadership ⇒ Ambiguity ⇒ Engagement ⇒ Well-being	−0.116	0.015	−0.121	−7.666	<.001
Leadership ⇒ Ambiguity ⇒ Well-being	−0.091	0.024	−0.094	−3.833	<.001
Leadership ⇒ Ambiguity ⇒ Teamwork ⇒ Engagement ⇒ Well-being	−0.019	0.003	−0.02	−5.996	<.001
Leadership ⇒ Autonomy ⇒ Teamwork ⇒ Engagement ⇒ Well-being	−0.278	0.025	−0.29	−10.949	<.001

Estimate: Unstandardized Regression Coefficient; SE: Standard Error; β: Standardized Beta Coefficient (indicating effect size); Z: Z-score for significance test. SE: Standard Error.

Teamwork showed a strong negative indirect effect on well-being via engagement (β = −0.448, *p* < .001). Similarly, autonomy and leadership demonstrated significant negative indirect effects on well-being through sequential pathways involving teamwork and engagement. Leadership also influenced well-being indirectly via coping with ambiguity alone and in combination with teamwork and engagement, underscoring its central role in shaping both job demands and resources within the model.

### Indirect effects (2023)

As shown in Table 5, the indirect effects observed in 2023 largely mirrored those found in 2022, although effect sizes were generally smaller. Coping with ambiguity continued to have a negative indirect effect on well-being through engagement (β = −0.140, *p* < .001) and through teamwork and engagement (β = −0.042, *p* < .001).

**Table 5:** Indirect effects between variables with 2023 data (significant effects).

Parameter	Estimate	SE	*β*	Z	*p*
Ambiguity ⇒ Engagement ⇒ Well-being	−0.092	0.025	−0.14	−3.729	<.001
Ambiguity ⇒ Teamwork ⇒ Engagement ⇒ Well-being	−0.04	0.005	−0.042	−7.526	<.001
Teamwork ⇒ Engagement ⇒ Well-being	−0.29	0.055	−0.346	−5.306	<.001
Autonomy ⇒ Teamwork ⇒ Engagement ⇒ Well-being	−0.132	0.028	−0.173	−4.661	<.001
Leadership ⇒ Teamwork ⇒ Engagement ⇒ Well-being	−0.131	0.025	−0.179	−5.338	<.001
Leadership ⇒ Ambiguity ⇒ Engagement ⇒ Well-being	−0.051	0.013	−0.067	−3.974	<.001
Leadership ⇒ Ambiguity ⇒ Well-being	−0.10	0.018	−0.133	−5.60	<.001
Leadership ⇒ Ambiguity ⇒ Teamwork ⇒ Engagement ⇒ Well-being	−0.019	0.003	−0.02	−5.996	<.001
Leadership ⇒ Autonomy ⇒ Teamwork ⇒ Engagement ⇒ Well-being	−0.278	0.025	−0.29	−10.949	<.001

Estimate: Unstandardized Regression Coefficient; SE: Standard Error; β: Standardized Beta Coefficient (indicating effect size); Z: Z-score for significance test.

SE: Standard Error.

Teamwork, autonomy, and leadership all demonstrated significant indirect effects on well-being via engagement-related pathways. Leadership again showed multiple indirect pathways affecting well-being, including routes through coping with ambiguity, teamwork, and engagement, confirming the robustness of these mediated relationships across time.

The relationship between group work and well-being, mediated by engagement, was empirically supported in the model test. Group work appears to be a major mediator of the relationships between autonomy and engagement, and between leadership and engagement. Factors such as autonomy and ambiguity coping in job demands also mediate the relationships between leadership and group work.

The graphic illustration of the model tested using the 2022 and 2023 samples is shown in Figs 1 and 2. The major contribution of these results lies in the empirical support for the relationships between leadership, group work, and work outcomes (engagement and well-being).

Group performance is significantly associated with leadership, which supports autonomy, a critical job resource such as a relevant personal resource, and the self-perception of the capacity to manage job ambiguity demands. By effectively managing these aspects, leadership fosters a work environment that promotes effective teamwork, thereby contributing to positive work outcomes. For instance, the beta coefficient of 0.502 for Leadership - > Teamwork in 2022 (Table 2) indicates that for every one-standard-deviation increase in effective leadership, teamwork is predicted to increase by approximately 0.502 standard deviations, representing a substantial and practically significant improvement in team dynamics. These results are detailed in Tables 4 and 5.

Despite using aggregated measures to test the model, the reliability indices were very positive, given the data from 2022 and 2023, which supports the inferences drawn from the research. These results are in Table 6.

**Table 6:** Reliability measures (2022 and 2023 measures).

Variable	α	⍵^1^	⍵^2^	⍵^3^	AVE
Leadership	0.979	0.979	0.979	0.979	0.923
Engagement	0.940	0.940	0.940	0.902	0.886
Well-being	0.971	0.972	0.972	0.947	0.920
Teamwork	0.963	0.965	0.965	0.967	0.875
Autonomy	0.954	0.969	0.972	0.972	0.915
Ambiguity	0.893	0.912	0.914	0.914	0.872

AVE: Average Variance Extracted. α = Cronbach’s Alpha; ⍵ = MC Donald’s ômega.

The reliability measures indicate that all the variables analysed have demonstrated excellent internal consistency (high Cronbach’s Alpha values, ranging from 0.940 to 0.979, and the Ômega reliability measures ranging from 0.902 to 0.979). In addition, the AVE values were high, all above 0.875, suggesting that a substantial portion of the variance may be explained by the items that make up each variable. These results indicate that the instruments used are highly reliable for measuring the variables in question.

## Discussion

This study examined how leadership, teamwork, autonomy, job ambiguity management, well-being, and engagement interact within NHS healthcare teams, using empirical data from 2022 and 2023. The central aim was to clarify how leadership and workplace dynamics drive team effectiveness and staff well-being.

Building on this central aim, the results confirmed H1, demonstrating a significant positive relationship between teamwork and work outcomes, particularly engagement. In the current study, teamwork was a significant predictor of work engagement in both the 2022 and 2023 analyses. These findings corroborate the literature, such as Xyrichis and Ream [15], which highlights that teamwork in healthcare settings may improve coordination and communication among professionals, leading to higher engagement and job satisfaction. Additionally, previous studies [10–12,14,31] have presented that effective teams, supported by HRM practices, have a direct impact on organisational performance, employee attitudes, and overall performance outcomes, as also emphasised by Richter et al. [71].

H2, which proposes a positive relationship between teamwork and well-being, was confirmed; however, this link is nuanced. Teamwork, as a job resource, supports engagement and well-being by providing social support, but well-being is also influenced by coping with ambiguity and limited autonomy. High job demands and insufficient resources, especially in intensive team-based NHS settings, can create strain, as the JD-R model suggests: teamwork helps, but only when balanced with autonomy and clear roles.

In light of the previous discussion, coping with ambiguity represents mechanisms through which individuals mobilize resources in response to ambiguity. Problem-focused control coping aligns with the JD-R assumption that proactive resource investment mitigates job demands, whereas reliance on emotion-focused support coping may indicate prolonged exposure to unresolved demands that exceed available resources [36]. From this perspective, coping with ambiguity reflects the dynamic interplay between job demands, personal resources, and regulatory strategies, consistent with the core assumptions of the JD-R model.

Continuing this line of inquiry, Hypothesis 3 (H3), which postulated that engagement would mediate the relationship between teamwork and well-being, was confirmed. Engagement acts as a significant mediator in this relationship, reinforcing the notion that work engagement, driven by teamwork, may mitigate negative effects on well-being, even in high-demand, complex settings. This finding aligns with Parr et al. [72], who identified engagement as a crucial factor in improving care quality and patient satisfaction, which in turn affects the well-being of healthcare professionals. Additionally, it is consistent with motivation and well-being theories that highlight engagement’s central role as a protective factor in the workplace [73].

Results for H4 and H5 show that leadership strongly predicts teamwork, with this relationship mediated by autonomy and ambiguity. Effective leaders align team and organizational goals, promote team cohesion, facilitate autonomy, and reduce role ambiguity [73–76], key for engagement and performance under the JD-R framework, where autonomy is a vital resource and ambiguity is a demand to be managed [44,45,77].

Finally, Hypothesis H6, which proposed that leadership would be associated with teamwork through an indirect relationship mediated by the skill to manage task ambiguity, was confirmed. Task ambiguity might hinder team effectiveness, but leadership that reduces it can significantly improve employee well-being. These results suggest that leaders who can minimise role ambiguity are essential in promoting a collaborative and healthy work environment, as evidenced by Weller et al. [78]. Leaders who clarify expectations and responsibilities not only reduce stress and increase job satisfaction but also facilitate teamwork, aligning with previous studies [79,80].

Overall, the findings of this study corroborate the literature on the importance of leadership and teamwork in healthcare settings. Studies developed by West et al. [73] and Ogbonnaya et al. [65] show that effective leadership and teamwork practices are associated with better organisational outcomes, including reduced patient mortality and improved employee well-being. Moreover, research by Valentine et al. [81] highlights the importance of using validated instruments to measure teamwork, which was considered in this study by utilising data from the NHS Staff Survey.

### Theoretical contributions

This study makes several unique theoretical contributions by applying and extending the JD-R model in the specific context of the NHS. First, it empirically validates the JD-R framework by demonstrating that leadership, as a critical job resource, is directly associated with teamwork and indirectly affects employee engagement and well-being through the mediation of both autonomy (a job resource) and ambiguity (a job demand). This provides a more nuanced understanding of the pathways through which leadership behaviors translate into team and individual outcomes. Secondly, the study highlights the dual nature of teamwork’s impact on well-being, showing that while it generally acts as a resource, its benefits may be undermined by high job demands, such as ambiguity, or by insufficient resources, such as autonomy. This enriches the JD-R model by illustrating the complex interplay between demands and resources within team contexts, particularly in high-pressure environments like health care. Finally, by explicitly modeling the mediating roles of autonomy and ambiguity, this research clarifies the mechanisms through which leadership might either buffer demands or enhance resources, thereby contributing to a more comprehensive theoretical understanding of organizational behavior in healthcare.

### Implications

The current study also has important practical implications for healthcare management. Firstly, the strong link between leadership and teamwork effectiveness suggests healthcare organisations should invest strategically in leadership development programmes that focus on enhancing team dynamics and, crucially, on fostering specific leadership styles that align with the JD-R model, such as transformational and participative leadership. Transformational leaders, by inspiring and empowering their teams, can enhance job resources like autonomy and foster a sense of purpose. Participative leadership, by involving employees in decision-making, promotes direct autonomy and reduces ambiguity by clarifying roles and expectations. Once fostering a leadership style that promotes autonomy and clarity, organisations might improve both employee engagement and well-being.

Secondly, the positive impact of well-structured teams on employee outcomes highlights the need for healthcare managers to prioritise the formation and maintenance of such teams. This involves not only assembling teams with the right mix of skills but also helping these teams to get clear goals, regular communication, and support from leadership. The finding that engagement mediates the relationship between teamwork and well-being suggests that interventions aimed at boosting engagement could have a dual benefit: improving overall job satisfaction and reducing burnout. Finally, given the association between task ambiguity and negative work outcomes, healthcare organisations should implement processes and systems that provide clear guidelines and reduce role ambiguity. Potential barriers to implementing such interventions in the NHS or broader healthcare contexts include resource constraints, hierarchical structures, staff shortages, high workloads that prevent participation in development programs, resistance to change, and ingrained organizational culture. Strategies to overcome these barriers could include phased implementation, pilot programs, securing leadership buy-in, and integrating development into existing structures.

Our results have practical relevance. Specifically, we recommend: (1) providing training interventions for leaders of teams with lower well-being and health scores to help them reduce the negative effects of excessive or conflicting work demands, foster psychological safety, and strengthen teamwork; and (2) including action plans in the NHS that invest in developing staff’s personal coping resources. This should be achieved through training, forums, and workshops that facilitate experience-sharing among teams and organizations with higher health and well-being scores, highlighting best practices for managing crises and work demands.

According to Wallbank [10], the 2022 NHS Survey results indicated dissatisfaction with salaries and declining workforce morale. However, in 2023, the NHS survey showed that approximately one-third of the workforce reported not feeling heard or supported by peers. They did not feel connected to their team and felt exhausted by work. Just less than half of participants reported that their organizations took action to improve workforce health and well-being, according to Oliver. Further studies are needed to investigate why actions undertaken since the release of the NHS Long Term Workforce Plan have not been effective in improving NHS staff well-being and performance indicators.

### Limitations and future research

This study presents limitations that should be acknowledged, which also open avenues for future research. Firstly, the cross-sectional design limits the ability to infer causality between the variables analysed. Future research should employ longitudinal designs to better understand the dynamic interaction and causal pathways between leadership, teamwork, autonomy, ambiguity, engagement, and well-being over time. Secondly, the analysis relied on aggregated data from the NHS Staff Survey, which, while providing a broad overview, may introduce ecological fallacy and limit the generalizability of individual-level inferences. Given the hierarchical nature of organizational data (individuals nested within groups/teams), future studies also could benefit from multilevel modeling to simultaneously analyze individual-level perceptions and group-level dynamics, thereby overcoming the limitations of aggregated data.

Furthermore, incorporating more comprehensive measures that consider positive aspects of well-being, such as life satisfaction and resilience, could provide more detailed insights. In addition, studies that stretch the notion of healthcare teams to include family members, involved in person or virtually, may help advance understanding of the effects on family outcomes [82,83]. Specific research questions for future inquiry include: i) How do different types of NHS teams (e.g., surgical vs. community nursing teams) experience and manage job demands (like ambiguity) and job resources (like autonomy) within the JD-R framework? ii) What is the impact of specific leadership development interventions on team-level job resources and demands, and subsequently on employee well-being and patient outcomes? iii) Can the proposed model be cross-culturally tested in different healthcare systems to assess the generalizability of these findings? iv) What is the role of additional personal resources (e.g., self-efficacy, resilience) or job demands (e.g., emotional demands, workload) within the JD-R structure in the NHS context? v) How are these dynamics related to direct patient outcomes, providing valuable insights beyond employees?

Finally, the results are based on data from the NHS in England, which may limit generalizability to other healthcare contexts or geographies. Comparative studies across healthcare systems would be valuable for testing the robustness of the findings reported in the present study.

## Conclusion

This study contributes uniquely to the organizational behavior and healthcare management literature by empirically testing a comprehensive model that integrates the JD-R model to explain the complex relationships between leadership, teamwork, autonomy, ambiguity, engagement, and well-being in the NHS. Specifically, it highlights how leadership, as a critical job resource, can mitigate job demands associated with ambiguity and foster the job resource of autonomy, thereby enhancing teamwork and subsequent positive work outcomes. While teamwork generally has a positive association with work outcomes, its impact on well-being is nuanced: high job demands, such as role ambiguity, and insufficient job resources, such as autonomy, can undermine these benefits, consistent with the JD-R framework’s health impairment process. This study also reinforces the importance of engagement as a mediator between teamwork and well-being, emphasizing its role in the motivational process.

For NHS policy, these findings underscore the imperative to invest strategically in leadership development programs that foster autonomy and clarify roles, alongside initiatives that actively promote well-structured, well-supported teamwork. Such interventions are crucial for increasing staff engagement, improving well-being, and ultimately optimizing healthcare service delivery within the unique demands of the NHS, contributing to a more sustainable workforce. These recommendations aim to create a work environment in which job resources are maximized and job demands are effectively managed, thereby improving employee and organizational outcomes.
