# Supplementary material for: Leadership, teamwork and work outcomes in healthcare: A test of a model
Source: PLoS One. 2026 Jul 10;21(7):e0353609. doi: 10.1371/journal.pone.0353609 (PMC13354071; doi:10.1371/journal.pone.0353609)
Supplement: Attachment — Submitted filename: Response to Reviewers.docx [file pone.0353609.s002.docx]

Dear Editors and Reviewers,

Thank you for the opportunity to revise our manuscript, “Leadership, teamwork and work outcomes in healthcare: a test of a model” (PONE-D-25-10310). We appreciate the thoughtful and constructive comments from both reviewers and the academic editor, which have significantly helped us strengthen the theoretical foundation and clarity of our work. We believe the revised version fully addresses the points raised and meets the publication criteria for PLOS ONE.

Below is our detailed point-by-point response to each comment.

**Journal Requirements**

1. **PLOS ONE Style Requirements:** The manuscript has been revised to adhere to all PLOS ONE style and formatting requirements, including the format for headings, authors, affiliations, and file naming conventions.
2. **Data Availability Statement (DAS):** We have strengthened the DAS to reflect the nature and availability of the data more accurately and completely. The aggregated dataset, which is the “minimal dataset” required to replicate this study’s analyses, will be provided as a Supporting Information file. We have also included a detailed statement on how researchers can request access to the original data, in line with PLOS ONE’s guidelines for third-party data.

**Reviewer #1**

**Response to Reviewer 1 Comments**

Reviewer 1 raised a fundamental criticism about the absence of a theoretical framework. We fully agree with this point, and the manuscript’s revision has been focused on addressing this issue. The **Job Demands-Resources (JD-R) model** has now been explicitly integrated throughout the manuscript to provide the necessary theoretical lens for our research model.

- **Theoretical Foundation:** The JD-R Model is now the central theory of the manuscript, justifying the selection of variables, the development of hypotheses, and the structure of the model.
- **Approach in the Text:** The JD-R Model is introduced in the abstract, with variables (autonomy, ambiguity) explicitly defined as a “key job resource” and a “significant job demand”. A new paragraph has been added to the introduction to articulate the literature gap from the JD-R perspective.

**Consequence for the Study:** The theoretical restructuring has allowed us to clearly articulate:

- The literature gap the study addresses (Page 3, lines 45-51).
- The conceptual innovation it introduces (Page 18, lines 383-397).
- The theoretical implications for future research and practice (Page 19, lines 415-433).

**Terminology and Hypotheses:** The use of terms like "model test" is now explicitly linked to the JD-R Model’s predictions, and each hypothesis has a detailed, theoretically informed rationale (Page 6-8, lines 108-155).

**Reviewer #2**

**Response to Reviewer 2 Comments**

We appreciate the detailed, section-by-section feedback, which was extremely helpful in improving the manuscript’s clarity and rigor.

**Abstract:**

- **Comment:** "Clarify the definitions of autonomy and ambiguity, highlighting their unique contributions and theoretical implications."
- **Response:** The abstract has been revised to explicitly define autonomy as a “key job resource” and ambiguity as a “significant job demand”. The role of the JD-R Model is also explicitly introduced to provide the theoretical context.

**Introduction:**

- **Comment:** "Better articulate the literature gaps and justify the mediators (autonomy, ambiguity). Make a smooth transition from the relevance of teamwork to the study objectives, addressing shortcomings of previous research."
- **Response:** A new paragraph was added to the introduction to clarify the literature gap from a JD-R perspective, explaining how the study fills this gap by investigating mediated relationships (Page 3, lines 45-51).

**Background:**

- **Comment:** "Justify the choice of autonomy and ambiguity as mediators, linking them to theory or empirical gaps. Add specific NHS context or examples for relevance."
- **Response:** The background section has been expanded to define JD-R, justifying the conceptualization of autonomy as a job resource (Page 5, lines 91-93) and ambiguity as a job demand (Page 4, lines 83-84), both aligned with the JD-R Model. Specific examples related to the NHS context have also been included to provide relevance (Page 5, lines 86-88).

**Study Hypotheses:**

- **Comment:** "Explicitly describe the causality and rationale for the hypotheses. Simplify the hypotheses for better readability; consider a figure that summarizes the relationships."
- **Response:** The hypotheses section has been completely rewritten. Each hypothesis now includes a detailed rationale based on the JD-R Model, explaining the expected causality (Page 6-8, lines 114-155). The manuscript also includes conceptual figures that summarize the relationships (Page 13, Fig 1 and Page 14, Fig 2 [Pages 33 and 34]).

**Materials and Methods - Participants:**

- **Comment:** "Provide more details on sample characteristics (roles, experience)."
- **Response:** The participants section was revised to provide more details on the composition of the 564 professional groups, clarifying that they represent a wide range of roles within the NHS, including nurses, doctors, allied health professionals, and administrative staff (Page 8, lines 16-167 and 172-173). The anonymized nature of the data was also made explicit (Page 8, lines 171-174).

**Measures:**

- **Comment:** "Address the limitations of the two-item ambiguity measure. Justify the focus on negative well-being measures and discuss potential biases."
- **Response:** The measures section was revised to include a statement acknowledging the limitations of the two-item measure for ambiguity (Page 11, lines 219-222). The focus on negative well-being is explicitly justified by its relevance to the “health impairment process” of the JD-R Model (Page 10, lines 211-215).

**Procedures:**

- **Comment:** "Justify the group-level analysis and its implications for validity and interpretation."
- **Response:** The text was expanded to justify the choice of group-level analysis, explaining that this approach was necessary due to the nature of the secondary data and the focus on organizational-level dynamics (Page 11, lines 230-235).

**Statistical Analysis:**

- **Comment:** "Explain the rationale for Robust Diagonally Weighted Least Squares (RDWLS). Clarify how the 2021 EFA informed the current CFA and SEM."
- **Response:** The statistical analysis section was revised to include the justification for using RDWLS, explaining its suitability for ordinal data and large sample sizes (Page 12, lines 248-252). The relationship between the EFA from 2021 and the CFA/SEM from 2022 was also clarified (Page 10/11, lines 240-245).

**Results:**

- **Comment:** "Discuss the reasons for the high RMSEA values and their implications. Use footnotes in tables to explain abbreviations and improve interpretability. Highlight practical significance alongside statistics."
- **Response:** The results section was revised to discuss the high RMSEA values, explaining that they can be inflated in complex models with large sample sizes (Page 12, lines 258-264). The practical significance of the beta coefficient for Leadership -> Teamwork was highlighted as a substantial improvement (Page 14, lines 295-297). All tables have been reviewed for clarity and footnotes have been added.

**Discussion:**

- **Comment:** "Elaborate on the complexity of the relationship between teamwork and well-being, emphasizing specific NHS implications. Discuss why autonomy and ambiguity work differently. Clearly separate theoretical contributions from practical implications."
- **Response:** The discussion was significantly revised. The complexity of the teamwork-well-being relationship is now explained through the balance of JD-R demands and resources, with specific NHS examples (Page 15/16, lines 331-339). The theoretical contributions (Page 18, lines 382-391) and practical implications (Page 18/19) have been separated into distinct subsections for greater clarity.

**Limitations and Future Research:**

- **Comment:** "Suggest methodological approaches (e.g., longitudinal, multi-level designs) to address the limitations. Propose specific research questions."
- **Response:** The section was revised to include suggestions for longitudinal and multilevel designs for future research. A list of specific research questions, which emerge from the identified limitations, has also been added to guide the future agenda (Page 20).

**Conclusion:**

- **Comment:** "Reinforce unique theoretical contributions. Add explicit NHS policy recommendations for practical impact."
- **Response:** The conclusion has been rewritten to clearly summarize the unique theoretical contribution of the study, which is the application and extension of the JD-R Model in the NHS context. Explicit and actionable policy recommendations for the NHS have been added with a focus on practical impact (Page 20, lines 431-447).

We thank you once again for your insightful review and comments.

Sincerely,

Elaine R. Neiva and Co-authors
