# Supplementary material for: Leadership, teamwork and work outcomes in healthcare: A test of a model
Source: PLoS One. 2026 Jul 10;21(7):e0353609. doi: 10.1371/journal.pone.0353609 (PMC13354071; doi:10.1371/journal.pone.0353609)
Supplement: Attachment — Submitted filename: Response_to_reviewers_auresp_2.docx [file pone.0353609.s003.docx]

**Dear Gede Juanamasta, Plos One Academic Editor,**

We sincerely thank the reviewers for their insightful comments. We have carefully revised the manuscript and addressed all points raised. The main changes are summarized below:

1. **Theoretical Anchoring** – The manuscript now provides a stronger analytical integration of the Job Demands–Resources (JD-R) model, with a reformulated theoretical foundation.
2. **Causality and Study Design** – Causal language has been removed, except for “mediated,” which is appropriate in correlational analyses.
3. **Measurement and Methodology** – The ambiguity variable was redefined to focus on task ambiguity. Justifications for group-level aggregation were expanded to include theoretical, practical, and statistical rationales, supported by the ICC (1), rwg, and ANOVA indices. Discussion of RMSEA now addresses model complexity, degrees of freedom, and sample size considerations.
4. **NHS Contextualization** – The manuscript now incorporates detailed contextual insights, including workforce structure, current pressures, and policy considerations.
5. **Practical Implications** – Specific interventions and policies for the NHS are now outlined, highlighting actionable leadership strategies.
6. **Discussion and Contribution** – The Discussion section was reorganized for clarity, distinguishing empirical results, theoretical implications, and practical contributions.
7. **Stylistic and Structural Improvements** – Parenthetical overuse was minimized, the abstract and conclusion were clarified, and all figures and tables now include explanatory notes and mediation labels.

We believe these revisions significantly enhance the manuscript and adequately address the reviewers’ concerns. We inform that the Revised Manuscript with Track Changes highlights text changes in red color.

Sincerely,

Dr. Elaine Rabelo Neiva

University of Brasilia
