# Supplementary material for: Leadership, teamwork and work outcomes in healthcare: A test of a model
Source: PLoS One. 2026 Jul 10;21(7):e0353609. doi: 10.1371/journal.pone.0353609 (PMC13354071; doi:10.1371/journal.pone.0353609)
Supplement: Attachment — Submitted filename: Response_to_Reviewers_R3.docx [file pone.0353609.s004.docx]

**Dear Gede Juanamasta, PLOS One Academic Editor,**

We sincerely thank the reviewer for the careful re-reading of our manuscript and the editor for the detailed revision requirements. We have addressed every concern raised in this round, including the two substantive comments from Reviewer #2, the journal-level requirements specified in the decision letter, and Reviewer #2’s response to the question on data availability. In the interest of full transparency, we also acknowledge below an inconsistency between our previous response letter and the text of the second revision, and we explain how it has now been resolved.

For ease of identification, all changes introduced in this third revision (R3) are highlighted in blue in the Revised Manuscript with Track Changes. Changes retained from the second revision remain in red. A clean version without any highlighting is also provided.

**Reviewer #2, point 1 — Ambiguity construct validity**

Comment: “Ambiguity construct validity”.

**Response:**

We agree that the brevity of the ambiguity scale warranted a more substantive defence of its construct validity. In the revised Measures subsection (Coping with Ambiguity), we now report the specific psychometric indicators for the two-item scale and add three conceptual arguments that support its use. The psychometric evidence, now also included as a dedicated row for “Ambiguity” in Table 6, comprises Cronbach's Alpha of 0.893, McDonald’s Omega between 0.912 and 0.914 across estimators, and an Average Variance Extracted of 0.872. These values imply standardised factor loadings above 0.90 and substantially exceed the conventional thresholds of 0.70 for reliability and 0.50 for convergent validity (Hair et al., 2019).

Beyond the numerical indicators, we have added three conceptual considerations to support construct validity. First, the scale is explicitly designed to capture a narrowly defined construct — coping with task ambiguity, understood as the perceived capacity to manage conflicting or contradictory work demands — rather than the broader and multidimensional role ambiguity construct in the tradition of Rizzo, House, and Lirtzman (1970). We have emphasised in the manuscript that the construct targeted is task ambiguity, not role ambiguity, and that this conceptual narrowness is coherent with a compact measurement. Second, we cite methodological research showing that short scales can achieve acceptable psychometric properties when the items are conceptually homogeneous, content-valid, and highly correlated (Postmes, Haslam, & Jans, 2013; Eisinga, Grotenhuis, & Pelzer, 2013; Fuchs & Diamantopoulos, 2009). Third, we note that the NHS Staff Survey items have been developed through more than two decades of iterative refinement and cognitive testing, supporting their content validity for the UK healthcare workforce. We have retained the acknowledgement, in the Limitations section, that a multi-item scale differentiating task and role ambiguity would represent an improvement and that this is a productive avenue for future research.

**Reviewer #2, point 2 — Aggregation justification (rwg/ANOVA vs. ICC/MAD discrepancy)**

Comment: “Aggregation justification, especially because the response letter promises rwg and ANOVA but the manuscript text shown reports ICC and MAD instead”.

**Response:**

We thank the reviewer for catching this inconsistency between our R2 response letter and the manuscript text, and we apologise for the lack of clarity it produced. We take full responsibility for the mismatch. The R2 response letter anticipated the reporting of r_wg(j)_ and ANOVA, but the final manuscript text only reported ICC and MAD, with no explicit justification for that methodological choice. We have now corrected this in three complementary ways, all visible in the Participants subsection of the revised manuscript.

First, we clarify that a one-way analysis of variance (ANOVA) was indeed conducted for each of the study variables, with the specific purpose of inspecting the variance decomposition and confirming the presence of meaningful between-group variance, a necessary precondition for justifying aggregation to the group level (Bliese, 2000). The ANOVA results confirmed statistically significant between-group variability across all constructs (*p* < 0.001), which supports the existence of systematic group-level effects in the data. This information is now explicitly reported in the manuscript.

Second, we provide a substantive methodological justification for having relied on the Median Absolute Deviation (MAD) rather than the r_wg(j)_ index. The MAD is conceptually analogous to the Average Deviation (AD) index proposed by Burke, Finkelstein, and Dusig (1999) and has been recommended as a robust alternative to r_wg(j)_ in the methodological literature (Burke & Dunlap, 2002; Dunlap, Burke, & Smith-Crowe, 2003; LeBreton & Senter, 2008). Our revised text explains the three main reasons we selected MAD: (i) unlike rwg(j), MAD is distribution-free and does not depend on an assumed null distribution, avoiding the well-documented problems of out-of-range and truncated values; (ii) MAD is less sensitive to variations in group size and rating scale characteristics, a relevant consideration given the heterogeneity of the NHS professional groups and variable respondents per trust; and (iii) MAD is expressed on the original metric of the response scale, providing a transparent and intuitive measure of within-group agreement. For a 5-point Likert scale, the established cutoff of 0.83 was met by the vast majority of teams in our sample.

Third, we now explicitly frame the aggregation decision as being supported by the convergence of three independent sources of evidence: (i) significant between-group ANOVA results confirming group-level variance; (ii) high and statistically significant ICC_(1)_ and ICC_(2)_ values indicating intragroup consistency; and (iii) MAD values below the recommended cutoff indicating within-group agreement. The revised Participants subsection now reads as a coherent statistical defence of the aggregation decision, and the references to Bliese (2000), Burke and Dunlap (2002), Dunlap et al. (2003), LeBreton and Senter (2008), and Burke et al. (1999) have been added to the reference list (refs. 75–79 in the revised manuscript).

**Reviewer #2, point 3 — Data availability (response moved from “No Response” in R2 to “No” in R3)**

Comment: Reviewer #2 marked “No” on the question “Have the authors made all data underlying the findings in their manuscript fully available?”

**Response:**

We thank the reviewer for surfacing this concern. We acknowledge that the previous Data Availability Statement could be read as ambiguous about the level of granularity at which the data are available, and we have now revised it to be fully compliant with the PLOS ONE Data Policy and to make the access pathways explicit. The study draws exclusively on secondary, anonymised data from the National NHS Staff Survey 2022 and 2023, which are aggregated at the level of NHS trust and are publicly available, without restriction or registration, from the official NHS Staff Survey portal at <https://www.nhsstaffsurveys.com/results/results-archive/>. There are no individual-level microdata underlying our analyses; the trust-level aggregates that we used are themselves the data underlying our findings. To further enhance reproducibility, the specific dataset used in our analyses, together with the variable derivation files, has been deposited in the Figshare data repository under the persistent DOI <https://doi.org/10.6084/m9.figshare.30114220>. The deposit is open access and citable, and any reader can directly reproduce the analyses reported in this manuscript from those files. The revised Data Availability Statement now states this distinction clearly, identifies the unit of analysis (NHS trust), and confirms that no restrictions apply to the data underlying the conclusions of the study.

**Additional changes**

In addition to the substantive revisions above, we have added a row for “Ambiguity” in Table 6 reporting Cronbach’s Alpha (0.893), McDonald’s Omega across estimators (ω¹ = 0.912, ω² = 0.914, ω³ = 0.914), and AVE (0.872), so that the psychometric indicators for the ambiguity coping construct are presented in parallel with the other latent constructs.

We thank the reviewer and the editor once again for their constructive feedback, which has substantially strengthened the methodological rigour of the paper. We believe these revisions significantly enhance the manuscript and adequately address the reviewers’ concerns. Revised Manuscript with Track Changes highlights text changes in red color.

Sincerely,

Dr. Elaine Rabelo Neiva

University of Brasília
