# Supplementary material for: Leadership, teamwork and work outcomes in healthcare: A test of a model
Source: PLoS One. 2026 Jul 10;21(7):e0353609. doi: 10.1371/journal.pone.0353609 (PMC13354071; doi:10.1371/journal.pone.0353609)
Supplement: Peer review history

# Peer review: Decision Letter 0

**DOI:** 10.1371/journal.pone.0353609.r001

**Authors:** I Gede Juanamasta

23 Mar 2025

PONE-D-25-10310

Leadership, teamwork and work outcomes in healthcare: a test of a model

PLOS ONE

Dear Dr. Neiva,

Thank you for submitting your manuscript to PLOS ONE. After careful consideration, we feel that it has merit but does not fully meet PLOS ONE’s publication criteria as it currently stands. Therefore, we invite you to submit a revised version of the manuscript that addresses the points raised during the review process.

Please submit your revised manuscript by May 07 2025 11:59PM. If you will need more time than this to complete your revisions, please reply to this message or contact the journal office at plosone@plos.org. When you're ready to submit your revision, log on to https://www.editorialmanager.com/pone/ and select the 'Submissions Needing Revision' folder to locate your manuscript file.

Please include the following items when submitting your revised manuscript:

- A rebuttal letter that responds to each point raised by the academic editor and reviewer(s). You should upload this letter as a separate file labeled 'Response to Reviewers'.
- A marked-up copy of your manuscript that highlights changes made to the original version. You should upload this as a separate file labeled 'Revised Manuscript with Track Changes'.
- An unmarked version of your revised paper without tracked changes. You should upload this as a separate file labeled 'Manuscript'.

If you would like to make changes to your financial disclosure, please include your updated statement in your cover letter. Guidelines for resubmitting your figure files are available below the reviewer comments at the end of this letter.

If applicable, we recommend that you deposit your laboratory protocols in protocols.io to enhance the reproducibility of your results. Protocols.io assigns your protocol its own identifier (DOI) so that it can be cited independently in the future. For instructions see: https://journals.plos.org/plosone/s/submission-guidelines#loc-laboratory-protocols. Additionally, PLOS ONE offers an option for publishing peer-reviewed Lab Protocol articles, which describe protocols hosted on protocols.io. Read more information on sharing protocols at https://plos.org/protocols?utm_medium=editorial-email&utm_source=authorletters&utm_campaign=protocols.

We look forward to receiving your revised manuscript.

Kind regards,

I Gede Juanamasta

Academic Editor

PLOS ONE

Journal requirements:

When submitting your revision, we need you to address these additional requirements.

1.  Please ensure that your manuscript meets PLOS ONE's style requirements, including those for file naming. The PLOS ONE style templates can be found at

https://journals.plos.org/plosone/s/file?id=wjVg/PLOSOne_formatting_sample_main_body.pdf  and

https://journals.plos.org/plosone/s/file?id=ba62/PLOSOne_formatting_sample_title_authors_affiliations.pdf

2. We note that your Data Availability Statement is currently as follows: [All relevant data are within the manuscript and its Supporting Information files.]

Please confirm at this time whether or not your submission contains all raw data required to replicate the results of your study. Authors must share the “minimal data set” for their submission. PLOS defines the minimal data set to consist of the data required to replicate all study findings reported in the article, as well as related metadata and methods (https://journals.plos.org/plosone/s/data-availability#loc-minimal-data-set-definition).

For example, authors should submit the following data:

- The values behind the means, standard deviations and other measures reported;

- The values used to build graphs;

- The points extracted from images for analysis.

Authors do not need to submit their entire data set if only a portion of the data was used in the reported study.

If your submission does not contain these data, please either upload them as Supporting Information files or deposit them to a stable, public repository and provide us with the relevant URLs, DOIs, or accession numbers. For a list of recommended repositories, please see https://journals.plos.org/plosone/s/recommended-repositories.

If there are ethical or legal restrictions on sharing a de-identified data set, please explain them in detail (e.g., data contain potentially sensitive information, data are owned by a third-party organization, etc.) and who has imposed them (e.g., an ethics committee). Please also provide contact information for a data access committee, ethics committee, or other institutional body to which data requests may be sent. If data are owned by a third party, please indicate how others may request data access.

[Note: HTML markup is below. Please do not edit.]

Reviewers' comments:

Reviewer's Responses to Questions

**Comments to the Author**

1. Is the manuscript technically sound, and do the data support the conclusions?

The manuscript must describe a technically sound piece of scientific research with data that supports the conclusions. Experiments must have been conducted rigorously, with appropriate controls, replication, and sample sizes. The conclusions must be drawn appropriately based on the data presented.

Reviewer #1: No

Reviewer #2: No

**********

2. Has the statistical analysis been performed appropriately and rigorously?

Reviewer #1: No

Reviewer #2: Yes

**********

3. Have the authors made all data underlying the findings in their manuscript fully available?

The PLOS Data policy requires authors to make all data underlying the findings described in their manuscript fully available without restriction, with rare exception (please refer to the Data Availability Statement in the manuscript PDF file). The data should be provided as part of the manuscript or its supporting information, or deposited to a public repository. For example, in addition to summary statistics, the data points behind means, medians and variance measures should be available. If there are restrictions on publicly sharing data—e.g. participant privacy or use of data from a third party—those must be specified.

Reviewer #1: No

Reviewer #2: No

**********

4. Is the manuscript presented in an intelligible fashion and written in standard English?

PLOS ONE does not copyedit accepted manuscripts, so the language in submitted articles must be clear, correct, and unambiguous. Any typographical or grammatical errors should be corrected at revision, so please note any specific errors here.

Reviewer #1: No

Reviewer #2: Yes

**********

5. Review Comments to the Author

Please use the space provided to explain your answers to the questions above. You may also include additional comments for the author, including concerns about dual publication, research ethics, or publication ethics. (Please upload your review as an attachment if it exceeds 20,000 characters)

Reviewer #1: A fundamental requirement of rigorous academic research—particularly in fields like organizational behavior and healthcare management—is that the proposed research model be grounded in an established theoretical framework. This paper presents a model linking leadership, teamwork, engagement, autonomy, and well-being but fails to anchor these relationships in any coherent or named theory, such as the Job Demands-Resources (JD-R) model.

Theory provides explanatory power. Without a theoretical lens, the paper becomes a collection of empirical associations, lacking depth, directionality, and conceptual robustness.

The absence of theory makes it unclear why these specific variables were selected, how they are expected to interact, or what new understanding the model offers beyond descriptive relationships.

Without a theoretical foundation, the study cannot claim to extend knowledge, test a theory, or even contribute to the ongoing scholarly discourse.

The authors use terminology like "model testing" and "mediating relationships" without defining what theory predicts or supports these mediation paths.

This results in a model that is atheoretical, generic, and not generalizable beyond the dataset used.

Why Theory Matters in Management Research:

Theoretical grounding distinguishes academic research from consultancy reports.

It guides variable selection, hypothesis development, and model structure.

It enables comparison with past research and accumulation of knowledge.

It justifies the study’s contribution to the literature.

Consequence:

Without a defined theoretical lens, the current study cannot demonstrate:

A gap in the literature it addresses,

A conceptual innovation it introduces, or

A theoretical implication for future research or practice.

Reviewer #2: Abstract

Clarify definitions of autonomy and ambiguity, highlighting their unique contributions and theoretical implications.

Introduction

Better articulate literature gaps and justify mediators (autonomy, ambiguity).

Smooth transition from teamwork relevance to study goals, addressing prior research shortcomings.

Background

Justify choice of autonomy and ambiguity as mediators, linking to theory or empirical gaps.

Add NHS-specific context or examples for relevance.

Study Hypotheses

Explicitly describe causality and rationale for hypotheses.

Streamline hypotheses for readability; consider a figure summarizing relationships.

Materials and Methods

Provide more detail on sample characteristics (roles, experience).

Clarify rationale for using group-level data and its methodological implications.

Measures

Address limitations of the two-item ambiguity measure.

Justify focus on negative well-being measures and discuss potential biases.

Procedures

Justify group-level analysis and its implications for validity and interpretation.

Statistical Analysis

Explain rationale for Robust Diagonally Weighted Least Squares (RDWLS).

Clarify how 2021 EFA informed current CFA and SEM.

Results

Discuss reasons for high RMSEA values and their implications.

Use table footnotes to explain abbreviations and improve interpretability.

Highlight practical significance alongside statistical significance.

Discussion

Elaborate on teamwork-well-being complexity, emphasizing NHS-specific implications.

Discuss why autonomy and ambiguity function differently (e.g., autonomy positive, ambiguity problematic).

Clearly separate theoretical contributions from practical implications.

Implications

Highlight specific leadership styles (e.g., transformational, participative) to target based on results.

Discuss potential barriers to implementing interventions in NHS or healthcare contexts.

Limitations and Future Research

Suggest methodological approaches (e.g., longitudinal, multi-level designs) to address limitations.

Propose specific research questions emerging from identified limitations (e.g., cross-cultural testing).

Conclusion

Reinforce unique theoretical contributions (e.g., leadership-ambiguity interplay in healthcare).

Add explicit NHS policy recommendations for practical impact.

References

Ensure citation consistency, especially for digital sources.

Tables and Figures

Add explanatory captions to figures for clarity.

Use clear table footnotes to explain abbreviations, scales, and statistics (e.g., RMSEA cutoffs).

**********

6. PLOS authors have the option to publish the peer review history of their article (what does this mean?). If published, this will include your full peer review and any attached files.

If you choose “no”, your identity will remain anonymous but your review may still be made public.

**Do you want your identity to be public for this peer review?**  For information about this choice, including consent withdrawal, please see our Privacy Policy.

Reviewer #1: No

Reviewer #2: **Yes:**  Yupin Aungsuroch

**********

[NOTE: If reviewer comments were submitted as an attachment file, they will be attached to this email and accessible via the submission site. Please log into your account, locate the manuscript record, and check for the action link "View Attachments". If this link does not appear, there are no attachment files.]

While revising your submission, please upload your figure files to the Preflight Analysis and Conversion Engine (PACE) digital diagnostic tool, https://pacev2.apexcovantage.com/. PACE helps ensure that figures meet PLOS requirements. To use PACE, you must first register as a user. Registration is free. Then, login and navigate to the UPLOAD tab, where you will find detailed instructions on how to use the tool. If you encounter any issues or have any questions when using PACE, please email PLOS at figures@plos.org. Please note that Supporting Information files do not need this step.

# Author comment: Author response to Decision Letter 1

**DOI:** 10.1371/journal.pone.0353609.r002

13 Sep 2025

Dear Editors and Reviewers,

Thank you for the opportunity to revise our manuscript, “Leadership, teamwork and work outcomes in healthcare: a test of a model” (PONE-D-25-10310). We appreciate the thoughtful and constructive comments from both reviewers and the academic editor, which have significantly helped us strengthen the theoretical foundation and clarity of our work. We believe the revised version fully addresses the points raised and meets the publication criteria for PLOS ONE.

Below is our detailed point-by-point response to each comment.

Journal Requirements

1. PLOS ONE Style Requirements: The manuscript has been revised to adhere to all PLOS ONE style and formatting requirements, including the format for headings, authors, affiliations, and file naming conventions.

2. Data Availability Statement (DAS): We have strengthened the DAS to reflect the nature and availability of the data more accurately and completely. The aggregated dataset, which is the “minimal dataset” required to replicate this study’s analyses, will be provided as a Supporting Information file. We have also included a detailed statement on how researchers can request access to the original data, in line with PLOS ONE’s guidelines for third-party data.

Reviewer #1

Response to Reviewer 1 Comments

Reviewer 1 raised a fundamental criticism about the absence of a theoretical framework. We fully agree with this point, and the manuscript’s revision has been focused on addressing this issue. The Job Demands-Resources (JD-R) model has now been explicitly integrated throughout the manuscript to provide the necessary theoretical lens for our research model.

• Theoretical Foundation: The JD-R Model is now the central theory of the manuscript, justifying the selection of variables, the development of hypotheses, and the structure of the model.

• Approach in the Text: The JD-R Model is introduced in the abstract, with variables (autonomy, ambiguity) explicitly defined as a “key job resource” and a “significant job demand”. A new paragraph has been added to the introduction to articulate the literature gap from the JD-R perspective.

Consequence for the Study: The theoretical restructuring has allowed us to clearly articulate:

• The literature gap the study addresses (Page 3, lines 45-51).

• The conceptual innovation it introduces (Page 18, lines 383-397).

• The theoretical implications for future research and practice (Page 19, lines 415-433).

Terminology and Hypotheses: The use of terms like "model test" is now explicitly linked to the JD-R Model’s predictions, and each hypothesis has a detailed, theoretically informed rationale (Page 6-8, lines 108-155).

Reviewer #2

Response to Reviewer 2 Comments

We appreciate the detailed, section-by-section feedback, which was extremely helpful in improving the manuscript’s clarity and rigor.

Abstract:

• Comment: "Clarify the definitions of autonomy and ambiguity, highlighting their unique contributions and theoretical implications."

• Response: The abstract has been revised to explicitly define autonomy as a “key job resource” and ambiguity as a “significant job demand”. The role of the JD-R Model is also explicitly introduced to provide the theoretical context.

Introduction:

• Comment: "Better articulate the literature gaps and justify the mediators (autonomy, ambiguity). Make a smooth transition from the relevance of teamwork to the study objectives, addressing shortcomings of previous research."

• Response: A new paragraph was added to the introduction to clarify the literature gap from a JD-R perspective, explaining how the study fills this gap by investigating mediated relationships (Page 3, lines 45-51).

Background:

• Comment: "Justify the choice of autonomy and ambiguity as mediators, linking them to theory or empirical gaps. Add specific NHS context or examples for relevance."

• Response: The background section has been expanded to define JD-R, justifying the conceptualization of autonomy as a job resource (Page 5, lines 91-93) and ambiguity as a job demand (Page 4, lines 83-84), both aligned with the JD-R Model. Specific examples related to the NHS context have also been included to provide relevance (Page 5, lines 86-88).

Study Hypotheses:

• Comment: "Explicitly describe the causality and rationale for the hypotheses. Simplify the hypotheses for better readability; consider a figure that summarizes the relationships."

• Response: The hypotheses section has been completely rewritten. Each hypothesis now includes a detailed rationale based on the JD-R Model, explaining the expected causality (Page 6-8, lines 114-155). The manuscript also includes conceptual figures that summarize the relationships (Page 13, Fig 1 and Page 14, Fig 2 [Pages 33 and 34]).

Materials and Methods - Participants:

• Comment: "Provide more details on sample characteristics (roles, experience)."

• Response: The participants section was revised to provide more details on the composition of the 564 professional groups, clarifying that they represent a wide range of roles within the NHS, including nurses, doctors, allied health professionals, and administrative staff (Page 8, lines 16-167 and 172-173). The anonymized nature of the data was also made explicit (Page 8, lines 171-174).

Measures:

• Comment: "Address the limitations of the two-item ambiguity measure. Justify the focus on negative well-being measures and discuss potential biases."

• Response: The measures section was revised to include a statement acknowledging the limitations of the two-item measure for ambiguity (Page 11, lines 219-222). The focus on negative well-being is explicitly justified by its relevance to the “health impairment process” of the JD-R Model (Page 10, lines 211-215).

Procedures:

• Comment: "Justify the group-level analysis and its implications for validity and interpretation."

• Response: The text was expanded to justify the choice of group-level analysis, explaining that this approach was necessary due to the nature of the secondary data and the focus on organizational-level dynamics (Page 11, lines 230-235).

Statistical Analysis:

• Comment: "Explain the rationale for Robust Diagonally Weighted Least Squares (RDWLS). Clarify how the 2021 EFA informed the current CFA and SEM."

• Response: The statistical analysis section was revised to include the justification for using RDWLS, explaining its suitability for ordinal data and large sample sizes (Page 12, lines 248-252). The relationship between the EFA from 2021 and the CFA/SEM from 2022 was also clarified (Page 10/11, lines 240-245).

Results:

• Comment: "Discuss the reasons for the high RMSEA values and their implications. Use footnotes in tables to explain abbreviations and improve interpretability. Highlight practical significance alongside statistics."

• Response: The results section was revised to discuss the high RMSEA values, explaining that they can be inflated in complex models with large sample sizes (Page 12, lines 258-264). The practical significance of the beta coefficient for Leadership -> Teamwork was highlighted as a substantial improvement (Page 14, lines 295-297). All tables have been reviewed for clarity and footnotes have been added.

Discussion:

• Comment: "Elaborate on the complexity of the relationship between teamwork and well-being, emphasizing specific NHS implications. Discuss why autonomy and ambiguity work differently. Clearly separate theoretical contributions from practical implications."

• Response: The discussion was significantly revised. The complexity of the teamwork-well-being relationship is now explained through the balance of JD-R demands and resources, with specific NHS examples (Page 15/16, lines 331-339). The theoretical contributions (Page 18, lines 382-391) and practical implications (Page 18/19) have been separated into distinct subsections for greater clarity.

Limitations and Future Research:

• Comment: "Suggest methodological approaches (e.g., longitudinal, multi-level designs) to address the limitations. Propose specific research questions."

• Response: The section was revised to include suggestions for longitudinal and multilevel designs for future research. A list of specific research questions, which emerge from the identified limitations, has also been added to guide the future agenda (Page 20).

Conclusion:

• Comment: "Reinforce unique theoretical contributions. Add explicit NHS policy recommendations for practical impact."

• Response: The conclusion has been rewritten to clearly summarize the unique theoretical contribution of the study, which is the application and extension of the JD-R Model in the NHS context. Explicit and actionable policy recommendations for the NHS have been added with a focus on practical impact (Page 20, lines 431-447).

We thank you once again for your insightful review and comments.

Sincerely,

Elaine R. Neiva and Co-authors

Attachment Submitted filename: Response to Reviewers.docx

# Peer review: Decision Letter 1

**DOI:** 10.1371/journal.pone.0353609.r003

**Authors:** I Gede Juanamasta, I Gede Juanamasta

18 Dec 2025

PONE-D-25-10310R1

Leadership, teamwork and work outcomes in healthcare: a test of a model

PLOS One

Dear Dr. Neiva,

Thank you for submitting your manuscript to PLOS ONE. After careful consideration, we feel that it has merit but does not fully meet PLOS ONE’s publication criteria as it currently stands. Therefore, we invite you to submit a revised version of the manuscript that addresses the points raised during the review process.

Please submit your revised manuscript by Feb 01 2026 11:59PM. If you will need more time than this to complete your revisions, please reply to this message or contact the journal office at plosone@plos.org. When you're ready to submit your revision, log on to https://www.editorialmanager.com/pone/ and select the 'Submissions Needing Revision' folder to locate your manuscript file.

Please include the following items when submitting your revised manuscript:

- A rebuttal letter that responds to each point raised by the academic editor and reviewer(s). You should upload this letter as a separate file labeled 'Response to Reviewers'.
- A marked-up copy of your manuscript that highlights changes made to the original version. You should upload this as a separate file labeled 'Revised Manuscript with Track Changes'.
- An unmarked version of your revised paper without tracked changes. You should upload this as a separate file labeled 'Manuscript'.

If you would like to make changes to your financial disclosure, please include your updated statement in your cover letter. Guidelines for resubmitting your figure files are available below the reviewer comments at the end of this letter.

If applicable, we recommend that you deposit your laboratory protocols in protocols.io to enhance the reproducibility of your results. Protocols.io assigns your protocol its own identifier (DOI) so that it can be cited independently in the future. For instructions see: https://journals.plos.org/plosone/s/submission-guidelines#loc-laboratory-protocols. Additionally, PLOS ONE offers an option for publishing peer-reviewed Lab Protocol articles, which describe protocols hosted on protocols.io. Read more information on sharing protocols at https://plos.org/protocols?utm_medium=editorial-email&utm_source=authorletters&utm_campaign=protocols.

We look forward to receiving your revised manuscript.

Kind regards,

I Gede Juanamasta

Academic Editor

PLOS One

**Journal Requirements:**

If the reviewer comments include a recommendation to cite specific previously published works, please review and evaluate these publications to determine whether they are relevant and should be cited. There is no requirement to cite these works unless the editor has indicated otherwise.

[Note: HTML markup is below. Please do not edit.]

Reviewers' comments:

Reviewer's Responses to Questions

**Comments to the Author**

1. If the authors have adequately addressed your comments raised in a previous round of review and you feel that this manuscript is now acceptable for publication, you may indicate that here to bypass the “Comments to the Author” section, enter your conflict of interest statement in the “Confidential to Editor” section, and submit your "Accept" recommendation.

Reviewer #2: All comments have been addressed

**********

2. Is the manuscript technically sound, and do the data support the conclusions?

The manuscript must describe a technically sound piece of scientific research with data that supports the conclusions. Experiments must have been conducted rigorously, with appropriate controls, replication, and sample sizes. The conclusions must be drawn appropriately based on the data presented.

Reviewer #2: Partly

**********

3. Has the statistical analysis been performed appropriately and rigorously?

Reviewer #2: No

**********

4. Have the authors made all data underlying the findings in their manuscript fully available?

The PLOS Data policy requires authors to make all data underlying the findings described in their manuscript fully available without restriction, with rare exception (please refer to the Data Availability Statement in the manuscript PDF file). The data should be provided as part of the manuscript or its supporting information, or deposited to a public repository. For example, in addition to summary statistics, the data points behind means, medians and variance measures should be available. If there are restrictions on publicly sharing data—e.g. participant privacy or use of data from a third party—those must be specified.

Reviewer #2: (No Response)

**********

5. Is the manuscript presented in an intelligible fashion and written in standard English?

PLOS ONE does not copyedit accepted manuscripts, so the language in submitted articles must be clear, correct, and unambiguous. Any typographical or grammatical errors should be corrected at revision, so please note any specific errors here.

Reviewer #2: No

**********

6. Review Comments to the Author

Please use the space provided to explain your answers to the questions above. You may also include additional comments for the author, including concerns about dual publication, research ethics, or publication ethics. (Please upload your review as an attachment if it exceeds 20,000 characters)

**Reviewer #2:**  1. Theoretical Anchoring: Surface-Level Use of JD-R

Although the revision now invokes the Job Demands–Resources (JD-R) model, the integration remains somewhat descriptive rather than analytical.

2. Causality and Study Design Ambiguity

The manuscript frequently uses causal language (“influences,” “mediates,” “leads to”) despite relying on cross-sectional data.

3. Measurement and Methodological Limitations

• The two-item ambiguity measure, though acknowledged as a limitation, still undermines construct validity.

• It may not capture task vs. role ambiguity distinctions, which are theoretically important.

• The justification for aggregating to group level remains thin.

• You mention rationale conceptually but provide no statistical indices (ICC(1), rwg, ANOVA tests) showing within-group agreement.

• You note and discuss elevated RMSEA but treat it descriptively.

• You don’t specify whether model complexity, small df, or sample size inflation is to blame.

4. NHS Contextualization: Still Superficial

The NHS references are present but limited to surface mentions (“in NHS settings,” “within healthcare teams”).

There’s little evidence of deep contextual insight—no mention of the NHS workforce model, pressures, or current policy debates.

5. Practical Implications: Too Generic

Although “transformational” and “participative” leadership are mentioned, there’s little specificity about what interventions or policies are feasible in the NHS.

6. Discussion and Contribution Clarity

The Discussion section still blends empirical summary, theory, and implications in long paragraphs.

7. Stylistic and Structural Issues

• Overuse of parentheticals (e.g., “(e.g., autonomy positive, ambiguity problematic)”) interrupts flow; rephrase narratively.

• Some sentences in Abstract and Conclusion are too dense. PLOS ONE favors clarity over compression.

• Figures are not fully self-explanatory. Add arrows, mediation labels, and clear notes (“all paths tested via SEM”).

**********

7. PLOS authors have the option to publish the peer review history of their article (what does this mean?). If published, this will include your full peer review and any attached files.

If you choose “no”, your identity will remain anonymous but your review may still be made public.

**Do you want your identity to be public for this peer review?**  For information about this choice, including consent withdrawal, please see our Privacy Policy.

Reviewer #2: **Yes:**  Yupin Aungsuroch

**********

[NOTE: If reviewer comments were submitted as an attachment file, they will be attached to this email and accessible via the submission site. Please log into your account, locate the manuscript record, and check for the action link "View Attachments". If this link does not appear, there are no attachment files.]

To ensure your figures meet our technical requirements, please review our figure guidelines: https://journals.plos.org/plosone/s/figures

You may also use PLOS’s free figure tool, NAAS, to help you prepare publication quality figures: https://journals.plos.org/plosone/s/figures#loc-tools-for-figure-preparation.

NAAS will assess whether your figures meet our technical requirements by comparing each figure against our figure specifications.

# Author comment: Author response to Decision Letter 2

**DOI:** 10.1371/journal.pone.0353609.r004

2 Feb 2026

Dear Gede Juanamasta, Plos One Academic Editor,

We sincerely thank the reviewers for their insightful comments. We have carefully revised the manuscript and addressed all points raised. The main changes are summarized below:

1. Theoretical Anchoring – The manuscript now provides a stronger analytical integration of the Job Demands–Resources (JD-R) model, with a reformulated theoretical foundation.

2. Causality and Study Design – Causal language has been removed, except for “mediated,” which is appropriate in correlational analyses.

3. Measurement and Methodology – The ambiguity variable was redefined to focus on task ambiguity. Justifications for group-level aggregation were expanded to include theoretical, practical, and statistical rationales, supported by the ICC (1), rwg, and ANOVA indices. Discussion of RMSEA now addresses model complexity, degrees of freedom, and sample size considerations.

4. NHS Contextualization – The manuscript now incorporates detailed contextual insights, including workforce structure, current pressures, and policy considerations.

5. Practical Implications – Specific interventions and policies for the NHS are now outlined, highlighting actionable leadership strategies.

6. Discussion and Contribution – The Discussion section was reorganized for clarity, distinguishing empirical results, theoretical implications, and practical contributions.

7. Stylistic and Structural Improvements – Parenthetical overuse was minimized, the abstract and conclusion were clarified, and all figures and tables now include explanatory notes and mediation labels.

We believe these revisions significantly enhance the manuscript and adequately address the reviewers’ concerns. We inform that the Revised Manuscript with Track Changes highlights text changes in red color.

Sincerely,

Dr. Elaine Rabelo Neiva

University of Brasilia

Attachment Submitted filename: Response_to_reviewers_auresp_2.docx

# Peer review: Decision Letter 2

**DOI:** 10.1371/journal.pone.0353609.r005

**Authors:** I Gede Juanamasta, I Gede Juanamasta, I Gede Juanamasta

20 Mar 2026

PONE-D-25-10310R2

Leadership, teamwork and work outcomes in healthcare: a test of a model

PLOS One

Dear Dr. Neiva,

Thank you for submitting your manuscript to PLOS ONE. After careful consideration, we feel that it has merit but does not fully meet PLOS ONE’s publication criteria as it currently stands. Therefore, we invite you to submit a revised version of the manuscript that addresses the points raised during the review process.

Please submit your revised manuscript by May 04 2026 11:59PM. If you will need more time than this to complete your revisions, please reply to this message or contact the journal office at plosone@plos.org. When you're ready to submit your revision, log on to https://www.editorialmanager.com/pone/ and select the 'Submissions Needing Revision' folder to locate your manuscript file.

Please include the following items when submitting your revised manuscript:

- A letter that responds to each point raised by the academic editor and reviewer(s). You should upload this letter as a separate file labeled 'Response to Reviewers'.
- A marked-up copy of your manuscript that highlights changes made to the original version. You should upload this as a separate file labeled 'Revised Manuscript with Track Changes'.
- An unmarked version of your revised paper without tracked changes. You should upload this as a separate file labeled 'Manuscript'.

-->If you would like to make changes to your financial disclosure, please include your updated statement in your cover letter. Guidelines for resubmitting your figure files are available below the reviewer comments at the end of this letter.

If applicable, we recommend that you deposit your laboratory protocols in protocols.io to enhance the reproducibility of your results. Protocols.io assigns your protocol its own identifier (DOI) so that it can be cited independently in the future. For instructions see: https://journals.plos.org/plosone/s/submission-guidelines#loc-laboratory-protocols. Additionally, PLOS ONE offers an option for publishing peer-reviewed Lab Protocol articles, which describe protocols hosted on protocols.io. Read more information on sharing protocols at https://plos.org/protocols?utm_medium=editorial-email&utm_source=authorletters&utm_campaign=protocols.

We look forward to receiving your revised manuscript.

Kind regards,

I Gede Juanamasta

Academic Editor

PLOS One

Journal Requirements:

1. If the reviewer comments include a recommendation to cite specific previously published works, please review and evaluate these publications to determine whether they are relevant and should be cited. There is no requirement to cite these works unless the editor has indicated otherwise.

2. Please review your reference list to ensure that it is complete and correct. If you have cited papers that have been retracted, please include the rationale for doing so in the manuscript text, or remove these references and replace them with relevant current references. Any changes to the reference list should be mentioned in the rebuttal letter that accompanies your revised manuscript. If you need to cite a retracted article, indicate the article’s retracted status in the References list and also include a citation and full reference for the retraction notice.

[Note: HTML markup is below. Please do not edit.]

Reviewers' comments:

Reviewer's Responses to Questions

**Comments to the Author**

1. If the authors have adequately addressed your comments raised in a previous round of review and you feel that this manuscript is now acceptable for publication, you may indicate that here to bypass the “Comments to the Author” section, enter your conflict of interest statement in the “Confidential to Editor” section, and submit your "Accept" recommendation.

Reviewer #2: All comments have been addressed

**********

2. Is the manuscript technically sound, and do the data support the conclusions?

The manuscript must describe a technically sound piece of scientific research with data that supports the conclusions. Experiments must have been conducted rigorously, with appropriate controls, replication, and sample sizes. The conclusions must be drawn appropriately based on the data presented.

Reviewer #2: Partly

**********

3. Has the statistical analysis been performed appropriately and rigorously?

Reviewer #2: Yes

**********

4. Have the authors made all data underlying the findings in their manuscript fully available?

The PLOS Data policy requires authors to make all data underlying the findings described in their manuscript fully available without restriction, with rare exception (please refer to the Data Availability Statement in the manuscript PDF file). The data should be provided as part of the manuscript or its supporting information, or deposited to a public repository. For example, in addition to summary statistics, the data points behind means, medians and variance measures should be available. If there are restrictions on publicly sharing data—e.g. participant privacy or use of data from a third party—those must be specified.

Reviewer #2: No

**********

5. Is the manuscript presented in an intelligible fashion and written in standard English?

PLOS ONE does not copyedit accepted manuscripts, so the language in submitted articles must be clear, correct, and unambiguous. Any typographical or grammatical errors should be corrected at revision, so please note any specific errors here.

Reviewer #2: Yes

**********

6. Review Comments to the Author

Please use the space provided to explain your answers to the questions above. You may also include additional comments for the author, including concerns about dual publication, research ethics, or publication ethics. (Please upload your review as an attachment if it exceeds 20,000 characters)

Reviewer #2: Ambiguity construct validity

Aggregation justification, especially because the response letter promises rwg and ANOVA but the manuscript text shown reports ICC and MAD instead

**********

7. PLOS authors have the option to publish the peer review history of their article (what does this mean?). If published, this will include your full peer review and any attached files.

If you choose “no”, your identity will remain anonymous but your review may still be made public.

**Do you want your identity to be public for this peer review?**  For information about this choice, including consent withdrawal, please see our Privacy Policy.

Reviewer #2: **Yes:**  Yupin Aungsuroch

**********

[NOTE: If reviewer comments were submitted as an attachment file, they will be attached to this email and accessible via the submission site. Please log into your account, locate the manuscript record, and check for the action link "View Attachments". If this link does not appear, there are no attachment files.]

To ensure your figures meet our technical requirements, please review our figure guidelines: https://journals.plos.org/plosone/s/figures

You may also use PLOS’s free figure tool, NAAS, to help you prepare publication quality figures: https://journals.plos.org/plosone/s/figures#loc-tools-for-figure-preparation.

NAAS will assess whether your figures meet our technical requirements by comparing each figure against our figure specifications.

# Author comment: Author response to Decision Letter 3

**DOI:** 10.1371/journal.pone.0353609.r006

4 May 2026

Dear Gede Juanamasta, PLOS One Academic Editor,

We sincerely thank the reviewer for the careful re-reading of our manuscript and the editor for the detailed revision requirements. We have addressed every concern raised in this round, including the two substantive comments from Reviewer #2, the journal-level requirements specified in the decision letter, and Reviewer #2’s response to the question on data availability. In the interest of full transparency, we also acknowledge below an inconsistency between our previous response letter and the text of the second revision, and we explain how it has now been resolved.

For ease of identification, all changes introduced in this third revision (R3) are highlighted in blue in the Revised Manuscript with Track Changes. Changes retained from the second revision remain in red. A clean version without any highlighting is also provided.

Reviewer #2, point 1 — Ambiguity construct validity

Comment: “Ambiguity construct validity”.

Response:

We agree that the brevity of the ambiguity scale warranted a more substantive defence of its construct validity. In the revised Measures subsection (Coping with Ambiguity), we now report the specific psychometric indicators for the two-item scale and add three conceptual arguments that support its use. The psychometric evidence, now also included as a dedicated row for “Ambiguity” in Table 6, comprises Cronbach's Alpha of 0.893, McDonald’s Omega between 0.912 and 0.914 across estimators, and an Average Variance Extracted of 0.872. These values imply standardised factor loadings above 0.90 and substantially exceed the conventional thresholds of 0.70 for reliability and 0.50 for convergent validity (Hair et al., 2019).

Beyond the numerical indicators, we have added three conceptual considerations to support construct validity. First, the scale is explicitly designed to capture a narrowly defined construct — coping with task ambiguity, understood as the perceived capacity to manage conflicting or contradictory work demands — rather than the broader and multidimensional role ambiguity construct in the tradition of Rizzo, House, and Lirtzman (1970). We have emphasised in the manuscript that the construct targeted is task ambiguity, not role ambiguity, and that this conceptual narrowness is coherent with a compact measurement. Second, we cite methodological research showing that short scales can achieve acceptable psychometric properties when the items are conceptually homogeneous, content-valid, and highly correlated (Postmes, Haslam, & Jans, 2013; Eisinga, Grotenhuis, & Pelzer, 2013; Fuchs & Diamantopoulos, 2009). Third, we note that the NHS Staff Survey items have been developed through more than two decades of iterative refinement and cognitive testing, supporting their content validity for the UK healthcare workforce. We have retained the acknowledgement, in the Limitations section, that a multi-item scale differentiating task and role ambiguity would represent an improvement and that this is a productive avenue for future research.

Reviewer #2, point 2 — Aggregation justification (rwg/ANOVA vs. ICC/MAD discrepancy)

Comment: “Aggregation justification, especially because the response letter promises rwg and ANOVA but the manuscript text shown reports ICC and MAD instead”.

Response:

We thank the reviewer for catching this inconsistency between our R2 response letter and the manuscript text, and we apologise for the lack of clarity it produced. We take full responsibility for the mismatch. The R2 response letter anticipated the reporting of rwg(j) and ANOVA, but the final manuscript text only reported ICC and MAD, with no explicit justification for that methodological choice. We have now corrected this in three complementary ways, all visible in the Participants subsection of the revised manuscript.

First, we clarify that a one-way analysis of variance (ANOVA) was indeed conducted for each of the study variables, with the specific purpose of inspecting the variance decomposition and confirming the presence of meaningful between-group variance, a necessary precondition for justifying aggregation to the group level (Bliese, 2000). The ANOVA results confirmed statistically significant between-group variability across all constructs (p < 0.001), which supports the existence of systematic group-level effects in the data. This information is now explicitly reported in the manuscript.

Second, we provide a substantive methodological justification for having relied on the Median Absolute Deviation (MAD) rather than the rwg(j) index. The MAD is conceptually analogous to the Average Deviation (AD) index proposed by Burke, Finkelstein, and Dusig (1999) and has been recommended as a robust alternative to rwg(j) in the methodological literature (Burke & Dunlap, 2002; Dunlap, Burke, & Smith-Crowe, 2003; LeBreton & Senter, 2008). Our revised text explains the three main reasons we selected MAD: (i) unlike rwg(j), MAD is distribution-free and does not depend on an assumed null distribution, avoiding the well-documented problems of out-of-range and truncated values; (ii) MAD is less sensitive to variations in group size and rating scale characteristics, a relevant consideration given the heterogeneity of the NHS professional groups and variable respondents per trust; and (iii) MAD is expressed on the original metric of the response scale, providing a transparent and intuitive measure of within-group agreement. For a 5-point Likert scale, the established cutoff of 0.83 was met by the vast majority of teams in our sample.

Third, we now explicitly frame the aggregation decision as being supported by the convergence of three independent sources of evidence: (i) significant between-group ANOVA results confirming group-level variance; (ii) high and statistically significant ICC(1) and ICC(2) values indicating intragroup consistency; and (iii) MAD values below the recommended cutoff indicating within-group agreement. The revised Participants subsection now reads as a coherent statistical defence of the aggregation decision, and the references to Bliese (2000), Burke and Dunlap (2002), Dunlap et al. (2003), LeBreton and Senter (2008), and Burke et al. (1999) have been added to the reference list (refs. 75–79 in the revised manuscript).

Reviewer #2, point 3 — Data availability (response moved from “No Response” in R2 to “No” in R3)

Comment: Reviewer #2 marked “No” on the question “Have the authors made all data underlying the findings in their manuscript fully available?”

Response:

We thank the reviewer for surfacing this concern. We acknowledge that the previous Data Availability Statement could be read as ambiguous about the level of granularity at which the data are available, and we have now revised it to be fully compliant with the PLOS ONE Data Policy and to make the access pathways explicit. The study draws exclusively on secondary, anonymised data from the National NHS Staff Survey 2022 and 2023, which are aggregated at the level of NHS trust and are publicly available, without restriction or registration, from the official NHS Staff Survey portal at https://www.nhsstaffsurveys.com/results/results-archive/. There are no individual-level microdata underlying our analyses; the trust-level aggregates that we used are themselves the data underlying our findings. To further enhance reproducibility, the specific dataset used in our analyses, together with the variable derivation files, has been deposited in the Figshare data repository under the persistent DOI https://doi.org/10.6084/m9.figshare.30114220. The deposit is open access and citable, and any reader can directly reproduce the analyses reported in this manuscript from those files. The revised Data Availability Statement now states this distinction clearly, identifies the unit of analysis (NHS trust), and confirms that no restrictions apply to the data underlying the conclusions of the study.

Additional changes

In addition to the substantive revisions above, we have added a row for “Ambiguity” in Table 6 reporting Cronbach’s Alpha (0.893), McDonald’s Omega across estimators (ω¹ = 0.912, ω² = 0.914, ω³ = 0.914), and AVE (0.872), so that the psychometric indicators for the ambiguity coping construct are presented in parallel with the other latent constructs.

We thank the reviewer and the editor once again for their constructive feedback, which has substantially strengthened the methodological rigour of the paper. We believe these revisions significantly enhance the manuscript and adequately address the reviewers’ concerns. Revised Manuscript with Track Changes highlights text changes in red color.

Sincerely,

Dr. Elaine Rabelo Neiva

University of Brasília

Attachment Submitted filename: Response_to_Reviewers_R3.docx

# Peer review: Decision Letter 3

**DOI:** 10.1371/journal.pone.0353609.r007

**Authors:** I Gede Juanamasta, I Gede Juanamasta, I Gede Juanamasta, I Gede Juanamasta

25 Jun 2026

<p>Leadership, teamwork and work outcomes in healthcare: a test of a model

PONE-D-25-10310R3

Dear Dr. Neiva,

We’re pleased to inform you that your manuscript has been judged scientifically suitable for publication and will be formally accepted for publication once it meets all outstanding technical requirements.

Within one week, you’ll receive an e-mail detailing the required amendments. When these have been addressed, you’ll receive a formal acceptance letter and your manuscript will be scheduled for publication.

An invoice will be generated when your article is formally accepted. Please note, if your institution has a publishing partnership with PLOS and your article meets the relevant criteria, all or part of your publication costs will be covered. Please make sure your user information is up-to-date by logging into Editorial Manager at Editorial Manager® and clicking the ‘Update My Information' link at the top of the page. For questions related to billing, please contact billing support.

If your institution or institutions have a press office, please notify them about your upcoming paper to help maximize its impact. If they’ll be preparing press materials, please inform our press team as soon as possible -- no later than 48 hours after receiving the formal acceptance. Your manuscript will remain under strict press embargo until 2 pm Eastern Time on the date of publication. For more information, please contact onepress@plos.org.

Kind regards,

I Gede Juanamasta

Academic Editor

PLOS One

Additional Editor Comments (optional):

Reviewers' comments:

Reviewer's Responses to Questions

**Comments to the Author**

1. If the authors have adequately addressed your comments raised in a previous round of review and you feel that this manuscript is now acceptable for publication, you may indicate that here to bypass the “Comments to the Author” section, enter your conflict of interest statement in the “Confidential to Editor” section, and submit your "Accept" recommendation.

Reviewer #2: All comments have been addressed

**********

2. Is the manuscript technically sound, and do the data support the conclusions?

The manuscript must describe a technically sound piece of scientific research with data that supports the conclusions. Experiments must have been conducted rigorously, with appropriate controls, replication, and sample sizes. The conclusions must be drawn appropriately based on the data presented.

Reviewer #2: Yes

**********

3. Has the statistical analysis been performed appropriately and rigorously?

Reviewer #2: Yes

**********

4. Have the authors made all data underlying the findings in their manuscript fully available?

The PLOS Data policy requires authors to make all data underlying the findings described in their manuscript fully available without restriction, with rare exception (please refer to the Data Availability Statement in the manuscript PDF file). The data should be provided as part of the manuscript or its supporting information, or deposited to a public repository. For example, in addition to summary statistics, the data points behind means, medians and variance measures should be available. If there are restrictions on publicly sharing data—e.g. participant privacy or use of data from a third party—those must be specified.

Reviewer #2: No

**********

5. Is the manuscript presented in an intelligible fashion and written in standard English?

PLOS ONE does not copyedit accepted manuscripts, so the language in submitted articles must be clear, correct, and unambiguous. Any typographical or grammatical errors should be corrected at revision, so please note any specific errors here.

Reviewer #2: Yes

**********

6. Review Comments to the Author

Please use the space provided to explain your answers to the questions above. You may also include additional comments for the author, including concerns about dual publication, research ethics, or publication ethics. (Please upload your review as an attachment if it exceeds 20,000 characters)

Reviewer #2: The authors have now provided a coherent methodological rationale and corrected the inconsistency between the response letter and manuscript. However, reporting would be stronger if the actual ANOVA and construct-specific aggregation statistics were presented rather than summarized

consistently refer to the construct as coping with ambiguity rather than ambiguity itself; and

provide a supplementary table with ANOVA, ICC(1), ICC(2), and MAD values for each aggregated construct.

**********

7. PLOS authors have the option to publish the peer review history of their article (what does this mean?). If published, this will include your full peer review and any attached files.

If you choose “no”, your identity will remain anonymous but your review may still be made public.

**Do you want your identity to be public for this peer review?**  For information about this choice, including consent withdrawal, please see our Privacy Policy.

Reviewer #2: **Yes:**  Yupin Aungsuroch

**********

# Editor report: Acceptance letter

**DOI:** 10.1371/journal.pone.0353609.r008

**Authors:** I Gede Juanamasta, I Gede Juanamasta, I Gede Juanamasta, I Gede Juanamasta

PONE-D-25-10310R3

PLOS One

Dear Dr. Neiva,

I'm pleased to inform you that your manuscript has been deemed suitable for publication in PLOS One. Congratulations! Your manuscript is now being handed over to our production team.

At this stage, our production department will prepare your paper for publication. This includes ensuring the following:

* All references, tables, and figures are properly cited

* All relevant supporting information is included in the manuscript submission,

* There are no issues that prevent the paper from being properly typeset

You will receive further instructions from the production team, including instructions on how to review your proof when it is ready. Please keep in mind that we are working through a large volume of accepted articles, so please give us a few days to review your paper and let you know the next and final steps.

Lastly, if your institution or institutions have a press office, please let them know about your upcoming paper now to help maximize its impact. If they'll be preparing press materials, please inform our press team within the next 48 hours. Your manuscript will remain under strict press embargo until 2 pm Eastern Time on the date of publication. For more information, please contact onepress@plos.org.

You will receive an invoice from PLOS for your publication fee after your manuscript has reached the completed accept phase. If you receive an email requesting payment before acceptance or for any other service, this may be a phishing scheme. Learn how to identify phishing emails and protect your accounts at https://explore.plos.org/phishing.

If we can help with anything else, please email us at customercare@plos.org.

Thank you for submitting your work to PLOS ONE and supporting open access.

Kind regards,

PLOS ONE Editorial Office Staff

on behalf of

Dr. I Gede Juanamasta

Academic Editor

PLOS One
